# Novel and cost-efficient design of stand-alone PV system with simulation using PVsyst and experimental validation

**DOI:** 10.1038/s41598-025-28401-y

**Published:** 2025-12-08

**Authors:** Ahmed Mashaly, Mohamed Elmadawy, Mohamed Elgohary, Ahmed SHAHIN

**Affiliations:** 1 Electrical Engineering Department, Faculty of Engineering, Mansoura University, 35516 Mansoura, Egypt; 2 Faculty of Engineering, Mansoura National University, Dakahlia, Egypt

**Keywords:** PVsyst, Photovoltaic (PV), PV design, Effective design, Performance ratio, PVsyst simulation, Energy science and technology, Engineering

## Abstract

Solar energy is gaining global prominence and is rapidly becoming a major energy source worldwide. According to reports, Egypt had made significant progress in solar energy installations by September 2022, reaching a total capacity of approximately 3.70 GW and setting renewable energy targets of 42% by 2035. Efficient and accurate PV system design is essential to meet future energy demands. This study presents a novel, cost-effective methodology for designing and validating a stand-alone photovoltaic (PV) system using PVsyst software, with a specific focus on evaluating the load requirements of the Solar Energy Lab at Mansoura University, located in the center of the Nile Delta, Egypt. A 2.64 kWp stand-alone system, integrated with a battery storage unit, is designed using PVsyst. The Lab’s annual energy demand is estimated at approximately 4279.78 kWh, while the system’s simulated generation reaches 4418.01 kWh, achieving a performance ratio (PR) of 0.81. PR analysis reveals seasonal variation, with January recording the highest value of 80% due to lower module temperatures, while June records the lowest at 76% as a result of higher temperatures. The annual average PR stands at 81%, with a levelized cost of energy (LCOE) of $0.082/kWh, indicating an optimized system design. The system’s performance is influenced by losses due to environmental factors such as dust, humidity, and temperature. A solar fraction of 87% reflects high reliability in meeting energy demand. To further enhance system efficiency, this study introduces a dynamic algorithm for system design, validated through simulations and a three-month experimental campaign using Watchpower software. The validated approach offers a scalable framework for academic institutions and facilities seeking to implement reliable, low-cost, off-grid PV systems in data-constrained environments.

## Introduction

The economic progress of any nation is dependent on its energy sources. With globalization and industrialization, the depletion of nonrenewable energy sources has become a pressing concern^1^. Countries worldwide are actively seeking alternative energy options, and among them, solar energy has emerged as a prominent solution^2^. Motivated by the need for decarbonization and the global energy crisis, the shift towards a green energy sector is of utmost importance^3^. The PV systems’ utilization plays a crucial role in mitigating global warming and achieving climate change objectives^4^. These systems have the ability to convert solar energy into electrical energy efficiently. Currently, solar systems are widely adopted as the preferred technology for harnessing solar energy^5^. One critical consideration is the storage of electrical energy derived from the sun to ensure a continuous supply during periods of low solar irradiation^6^. Various factors come into play in determining the effective utilization of solar energy, including geographical and weather conditions and the electrical load consumption^7^. Solar energy utilization is expanding across the globe due to its abundant availability. The sun alone possesses an interceptable potential energy surpassing the current human energy consumption requirements^8^. Furthermore, solar energy is a sustainable and environmentally friendly source that holds great potential to meet future energy demands^7^. To address these concerns, proactive measures have been implemented, including the ratification of the Paris Agreement by numerous governments, aimed at curbing $CO_{2}$ emissions, a significant contributor to global warming^9^. Over the past few decades, photovoltaic systems have undergone significant advancements in terms of technical efficiency, maintenance considerations, and grid-forming converters^10^. The performance of PV arrays is influenced by ambient conditions such as solar irradiance, wind, and temperature^11^. Consequently, the behavior of solar cells is significantly affected by voltage fluctuations caused by temperature changes and current fluctuations caused by variations in solar irradiation^12^. In this context, the implementation of effective control techniques is crucial to mitigate the impact of unpredictable weather conditions, ensuring the reliability and security of energy resources^13^. Egypt has significant potential to harness solar energy due to its geographical location. In 2023, a report released by the New and Renewable Energy Authority (NREA) revealed that Egypt had expanded its renewable energy capacity to 6500 MW for the country’s power generation, comprising 21% of the total peak power demand^14^. Approximately 35% of this capacity is attributed to private sector initiatives. The total energy production had increased to 25,100 GWh, comprising 15,000 GWh from hydroelectric sources, 5600 GWh from wind power, and 4,500 GWh from solar power^15^. The Egyptian government has established ambitious goals by 2035 to acquire 42% of the country’s electricity from renewable sources. Solar energy offers a wide range of applications beyond electrical energy, including water heating, room heating, solar pumps, and dryers^16^. To achieve these goals, two approaches can be taken: utilizing batteries for stand-alone systems or on-grid systems in which the solar power systems are connected to the main grid^17^. Stand-alone rooftop systems can greatly benefit households and medium-sized enterprises by reducing their peak loads. Evaluating the efficiency of photovoltaic systems involves considering important variables such as the solar yield, performance ratio, and system losses. The performance ratio, which relates the actual and theoretical outputs of solar energy while accounting for various losses, depends on many factors like ambient conditions, mounting systems, and electrical designs^18^. Utilizing coolants during high-temperature days can enhance the performance of solar cell modules by preventing overheating. Accurate location-specific parameters are crucial for designing, operating, maintaining, and sizing rooftop systems^19^. Several simulation software, such as PVsyst, INSEL, TRNSYS, PVSOL, and SOLARPRO, along with economic assessment tools like HOMER, Solar Advisor Model (SAM), RETScreen, SOLinvest, and Energy Periscope, are available to calculate energy production and assess the economic viability of PV systems^20^. Table 1 gives a comparison of the most used software; HOMER and SAM with proposed method for designing the PV stand-alone system. These software tools also aid in determining the performance ratio and minimizing losses. Meteorological databases from sources such as AEMET European Solar Radiation Atlas, NASA, METEONORM, ISPRA-GIS, HELIOS, Solaris, and PV-Design-Pro are used for simulations, with this study utilizing data from METEONORM and PVsyst simulations^21–23^. PVsyst is a widely employed tool for simulating stand-alone photovoltaic system performance. By example, reporting an annual array output energy of 841.31 kWh with 735.84 kWh supplied to the load^24^. In Odisha, India, with 1156.39 W/$\hbox {m}^2$ average solar irradiance, a 1 kWp rooftop PV system generates approximately 4.8 kWh/day, offsetting 28 tons of $CO_{2}$ over its lifetime. To validate system performance, a case study utilized PVsyst V6.84 to simulate a 2 kWp rooftop PV system for an 8.9 kWh/day residential load^25^. A PVsyst case study^22^ analyzed a stand-alone solar system in Bikaner, designed for an annual demand of 1086.24 kWh. Results show that 1143.6 kWh/year generated, with 1068.12 kWh supplied to the load slightly below demand due to various system losses. The annual performance ratio averaged 72.8% and between 64% and 86% monthly. This highlights performance analysis’s importance for optimal energy delivery and reliability in PV system design. This analysis quantified power losses, enabling optimal system sizing and demonstrating PVsyst’s reliability in forecasting energy delivery, which is critical for design optimization and efficiency assessment in PV applications. Despite some advances in PV modeling software, most studies lack experimental validation for systems designed with minimal instrumentation. Also, there have been limited academic studies addressing the problems of PV deployment by using streamlined tools like PVsyst. This study fills this gap by introducing a practical, experimentally verified methodology suited for educational Labs in the developing regions. The key contributions include simulation-based design optimization, low-cost hardware implementation, and real-time performance validation under actual climatic conditions.

The main objectives and contributions of this study are:

1. Developing an efficient design method for the PV stand-alone system based on simulation by software PVsyst and experiments.
2. Evaluate the solar energy potential at the selected location (Mansoura University, Egypt) based on actual meteorological data.
3. Identify the minimum load requirements to sustain the daily operation of the Solar Energy Lab as a stand-alone system.
4. Design a photovoltaic system layout using PVsyst software, optimized for local environmental conditions and practical implementation constraints.
5. Introduce a streamlined design methodology that avoids the use of costly pyranometers and multiple current sensors, relying instead on a single current measurement and simulation-based estimation—representing a key novelty of this work.
6. Simulate system performance using PVsyst, including detailed loss analysis, performance ratio (PR), and solar fraction (SF), to estimate the long-term behavior of the system.
7. Demonstrate that a reliable off-grid PV system can be deployed in institutional settings using minimal instrumentation and open-access tools—providing a practical model for similar environments in regions with limited technical infrastructure.
8. Assessing the performance ratio and losses of the PV system through simulation using PVsyst software.
9. Correlating the simulation and the experimental results for the selected site.
10. Experimentally validate the simulation model through real-world measurements of energy output, irradiance, and temperature under operating conditions, confirming the effectiveness of the proposed design approach.

**Table 1:** Comparison of PV stand-alone design approaches and proposed method (PM*).

Refs.	Design methodology	Tools/software	Validation approach	Key metrics	Remarks/gaps
^22^	Stand-alone PV design with thermal performance focus	PVsyst	Simulation only	PR, thermal losses	No experimental validation; focus on temperature effect.
^26^	Hybrid PV–diesel system sizing optimization	MATLAB, simulator	Simulation on rural case study	Cost, $CO_{2}$ emissions, unscheduled load	Strong optimization; lacks PR, LCOE, experimental validation.
^27^	Optimal sizing and battery management for off-grid PV	PVsyst, prototype	Simulation, Lab test	PR, autonomy, Battery SOC	Limited validation duration; lacks LCOE analysis.
^28^	Sizing stand-alone PV and battery for rural use	PVsyst	Simulation only	Load coverage, autonomy	No cost or performance validation.
^29^	Enhanced MPPT tracking for stand-alone PV system	MATLAB, PVsyst	Simulation, MPPT test	PR, MPPT response	Effective control logic; lacks cost metrics and long-term data.
^30^	Techno-economic sizing of PV–battery system in remote areas	HOMER Pro + MATLAB	Simulation + sensitivity analysis	LCOE, $CO_{2}$ savings	Strong optimization; lacks physical validation.
PM*	Dynamic PV sizing with real load and experimental validation	PVsyst, WatchPower	Real time, simulation	PR, SF, LCOE	Replicable, cost-effective, validated in academic off-grid case study.

## System description and modeling

**Fig. 1:**
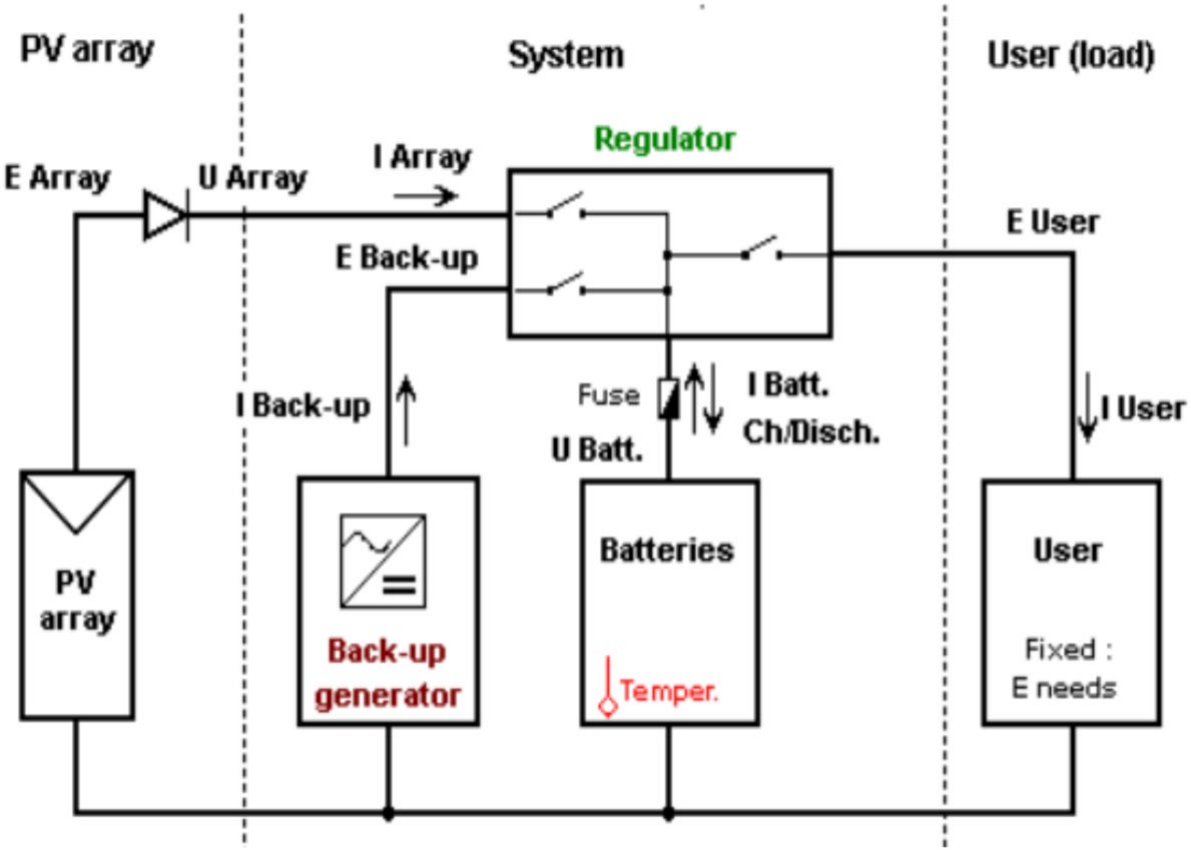
Layout of stand-alone system as designed by PVsyst.

**Fig. 2:**
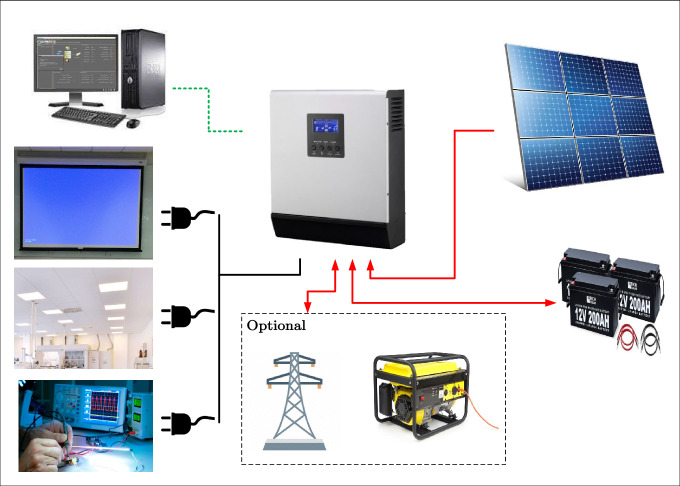
Configuration of a solar photovoltaic (PV) system, illustrating the interconnections between PV panels, an inverter, battery storage, various loads, and optional grid/generator inputs.

The efficiency of a solar system is enhanced by minimizing losses through component optimization, like selecting compatible inverters and ensuring module uniformity. Figure 1 represents the architecture of the proposed system operating under low-voltage (LV), low-power DC comprising the array feeding the DC loads by its own boost converter. The configuration of a solar photovoltaic (PV) system, illustrating the interconnections between PV panels, an inverter, battery storage, various loads, and optional grid/generator inputs, is shown in Fig. 2. The system mainly consists of arrays connected with the battery storage system through a battery charger with its own MPPT, then battery-stored energy is converted to AC for appliances, emphasizing the importance of mitigating losses in cable systems for optimal performance and maximizing power utilization. The system also includes a bypass diode for protection^31^. Additionally, UV-safe and weather-resistant cables are essential for these open-air applications.

This study focuses on analyzing the design and performance of a stand-alone solar photovoltaic system. The investigation explores losses attributed to various factors and closely monitors the plant’s performance using the performance ratio metric. Losses across different aspects are evaluated using PVsyst simulation. It is also used to calculate the performance ratio based on simulated performance. Additionally, the execution of the plant measures energy, solar resources, and the overall impact of the performance ratio and losses.

### Modeling of photovoltaic panels

**Fig. 3:**
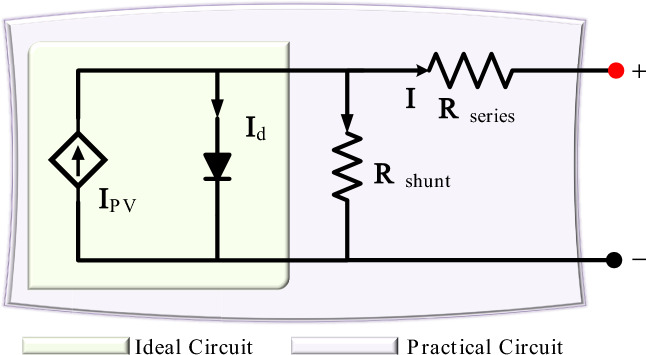
Equivalent model of solar cell.

The solar cell can be represented as shown in Fig. 3 with its basic model, which is detailed in^32^ by:

1 $$\begin{aligned} I = I_{pv}^{cell} - \underbrace{I_{rs}^{cell} \Big [ exp \Big ( \frac{qV_{pv}}{akT} \Big ) -1 \Big ]}_{I_d} \end{aligned}$$

To precisely simulate the practical behavior of arrays consisting of various attached cells, other parameters should be considered. This practical model is characterized by accuracy and simplicity; therefore, it is widely used. Accordingly, the array behavior can be defined by the model given by equations (2), (3), and (4) and as detailled in^32^.

2 $$\begin{aligned} I= & I_{pv}-I_{rs}\Big [ exp \Big ( \frac{V_{pv} +I r_{se}^{pv}}{aV_{th}}\Big ) -1 \Big ] - \frac{V_{pv}+I r_{se}^{pv}}{r_{p}^{pv}} \end{aligned}$$

3 $$\begin{aligned} I_{pv}= & \Big ( I_{pv}^{n}+k_{sc} \Delta T \Big ) GG_{n}^{-1} \end{aligned}$$

4 $$\begin{aligned} I_{rs}= & \frac{I_{sc}^{n}T_{n}^{3}T^{-3}}{exp\big ( V_{oc}^{n}a^{-1}V_{th}^{-1} \big )-1} exp \Big [ \frac{qW_g(T-T_n)}{akTT_n} \Big ] \end{aligned}$$

Table 2 lists the parameters of the solar panel as detailed in^31,32^ by the equivalent circuit of the solar cell as shown in Fig. 3.

**Table 2:** Solar array parameters.^31,32^.

Symbol	Value	Symbol	Value
$G_n$	1000 W/$\hbox {m}^2$	*a*	1.30
*q*	1.60e-19 C	$k_{sc}$	1.7e-3
*k*	1.3806503e-23 J/K	$T_n$	$25^\circ$C
$r_{pv}^{se}$	0.22 $\Omega$	$W_g$	1.7e-3
$r_{pv}^{p}$	415.40 $\Omega$	$P_{max}$	330 W
$V_{oc}$	45.50 V	$V_{MPP}$	37.10 V
$I_{sc}^n$	9.40 A	$I_{MPP}$	8.90 A

In some cases, the PV system operates under varying solar irradiance conditions throughout the day. This variation affects the output power, output voltage, and relationship of current under different temperature conditions. The solar module $I-V$ and $P-V$ characteristics under different solar irradiance and the corresponding maximum power are detailed in^31^. Selecting the appropriate panel is crucial for designing a system that meets the total load demand efficiently with minimal panel usage. The panel selection is influenced by the following factors: solar irradiance, temperature, voltage, current, and configuration.

### Methodology for designing PV system

When designing a photovoltaic system, the geographical location does not constrain the configuration but rather depends on solar irradiance levels. The quality of modules, inverters, and the orientation of solar panels are pivotal factors shaping a system’s design and efficiency, enabling adaptability across diverse settings. The methodology for crafting a PV system involves a comprehensive approach that delves into critical considerations. Solar irradiance levels at a specific site are foundational in gauging the system’s energy potential accurately. The methodology avoids the use of pyranometers and multiple sensors by employing a simulation-based estimation. The design is validated with a single-point current measurement and experimental data by real-time Watchpower based software, representing a novel approach for academic system design. horough comprehension analysis of these levels is crucial for precise performance estimations. Moreover, selecting components of superior quality tailored to site-specific demands is imperative for optimal performance. Additionally, the positioning and tilt of solar panels are pivotal design aspects directly impacting energy generation. Strategic alignment towards the sun and ideal tilt angles serve to maximize sunlight exposure, thereby boosting overall system performance. By intricately weaving together these elements throughout the design process, a meticulously planned methodology ensures the seamless deployment of a dependable and efficient photovoltaic system customized to meet the distinct requirements of each individual project.

### Application of PVsyst software

**Fig. 4:**
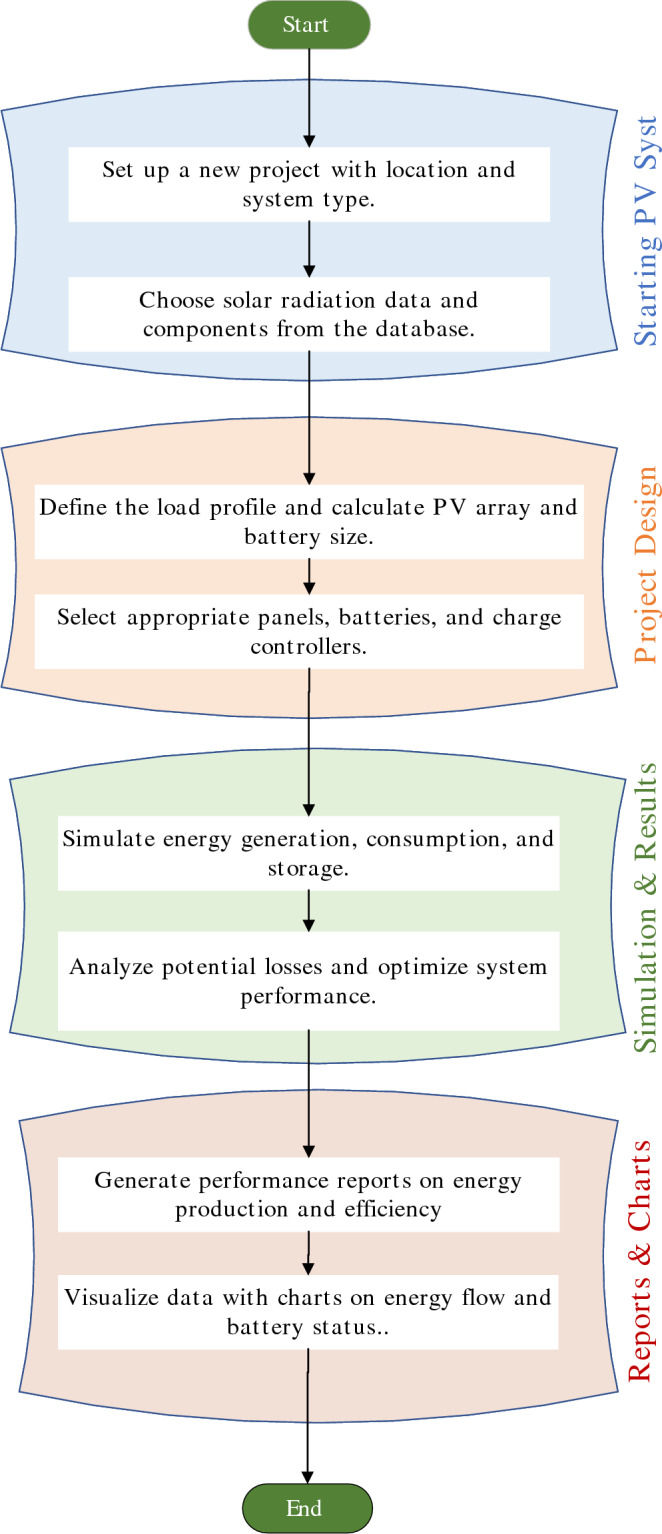
Functionality of PVsyst to design stand-alone system.

In designing the PV system, PVsyst V7.4, a PC software package, is configured using real site coordinates, METEONORM weather data, and detailed load profiles for the Solar Energy Lab. It customizes parameters for solar modules, enabling the study, sizing, and analysis of various systems such as grid-connected and stand-alone setups. The software incorporates comprehensive databases for meteorological and component data, along with general solar energy tools. It facilitates the design of system configurations and provides an estimation of the energy generated. Table 3 presents a summary of PVsyst setup and a comparison with HOMER and SAM software. The software relies on geographical information to simulate the system sizing accurately, as shown in Fig. 4. The outcomes of simulations conducted in PVsyst can encompass various variables, which can be presented in monthly, daily, or hourly values. One valuable feature is the “loss diagram,” which identifies potential weaknesses in the system design^26^. PVsyst offers a range of pre-existing sites and meteorological files in its databases, but users also have the option to create their own projects based on the specific location and meteorological data they intend to use. Design of the system is achieved by two steps; the first step is creating a system variant, and the second step is the simulation. In the first step, users define the calculation version of their project. They have the flexibility to specify the module orientation, system configuration, and loss parameters according to their requirements. The second step is running the simulation process to generate various graphs and reports that provide insights into the performance of the system. The simulation process in PVsyst involves a series of steps. Users can conveniently analyze the results within the PVsyst program, export them to other software for further analysis, or save them for future evaluation.

**Table 3:** Summary of PVsyst setup and comparison with HOMER and SAM software^33–35^.

Aspect	PVsyst Setup, contribution	HOMER and SAM
Primary focus	Technical simulation and validation of stand-alone or on-grid PV systems	HOMER: Hybrid system optimization with multi-source integration. SAM: Financial-performance modeling for various RE technologies.
Location and climatic data	Specific site coordinates; Meteonorm or measured data used	All tools support climate data. HOMER: Uses typical profiles. SAM: Integrates NREL database.
System configuration	Off/on-grid cases, diesel, pumping	HOMER: Supports hybrid grid/off-grid, diesel, wind. SAM: Highly customized system architectures.
Component sizing	Load-driven design (PV array, inverter, battery); validated with experiments	HOMER: Automated sizing via optimization. SAM: Sizing based on financial/technical return.
Load profile	Defined by end-use needs (e.g., Lab, rural household)	HOMER: Supports complex and variable loads. SAM: Load inputs used in financial forecasting.
Simulation outputs	PR, SF, LCOE, loss diagrams	HOMER: Cost and reliability optimization. SAM: Internal rate of return (IRR), net present value (NPV), LCOE, tariff impact.
Validation approach	Field data, user-defined data	HOMER: Typically scenario-based with assumed data. SAM: Used in post-design financial validation.
Optimization strategy	Iterative trade-off between cost, autonomy, and efficiency	HOMER: Algorithmic optimization for lowest NPC or LCOE. SAM: Optimization based on financial metrics.
Sensitivity analysis	Conducted by varying load, irradiance, battery size	HOMER: Extensive built-in sensitivity engine. SAM: Economic scenario testing (tariffs, incentives).
Financial modeling	Basic LCOE; no advanced financial returns modeling	HOMER: Cost-effectiveness focus. SAM: Advanced modeling: IRR, NPV, payback.
Applications	Research and experimental PV design validation in remote on/off-grid, pumping setups	HOMER: Multi-source hybrid design for microgrids. SAM: Investment-level feasibility studies.

### Geographical location and solar horizon

Determining the sunlight availability at a specific location is necessary for better design. It helps in planning and designing solar energy systems. The solar irradiance depends on the geographical location and the time of the year. The monthly values of global horizontal, diffused, extraterrestrial irradiation, clearness index, ambient temperature, wind velocity, etc. have been obtained by PVsyst and described in Table 4 for the selected location: Mansoura City, Egypt. Mansoura City lies between $31.03^{\circ }$N latitude and $31.38^{\circ }$E longitude. While temperature also plays a role, its impact is secondary to that of irradiance; cooler temperatures generally improve performance. Factors such as irradiance levels, ambient temperature, and wind speed influence the cell temperature. The available current and output power of a solar array depend directly on the amount of irradiance it receives.

**Fig. 5:**
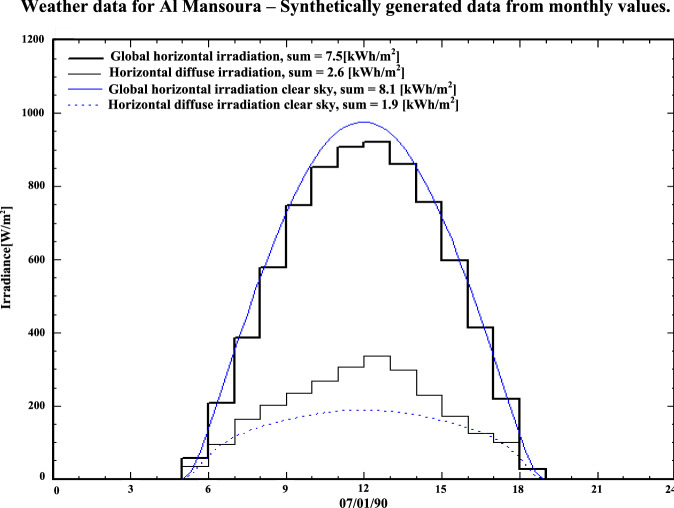
Irradiation components for Mansoura-one day.

**Fig. 6:**
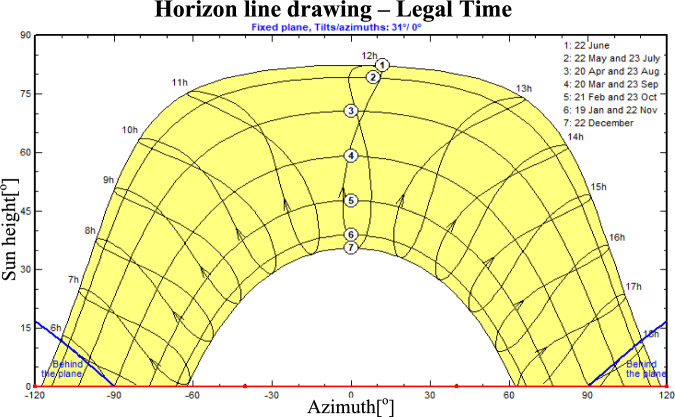
Sun paths height/azimuth plot for chosen site within PVsyst..

Figure 5 illustrates the variation of solar irradiance throughout the day, showing both direct and diffuse components. The solar data are some of the major inputs for an energy yield evaluation. The solar irradiance during the day is composed of direct and diffused irradiance, and its highest value is during midday time with the solar energy of a certain day. The analysis is generated by PVsyst and based on weather records, including temperature and humidity, at the selected locations. Figure 6 represents the annual solar horizon profile for Mansoura city. The accessible solar energy is depicted within the horizon boundary. Two slanted (oblique) blue lines appearing on either side of Fig. 6 represent the sunrise (left) and sunset (right) periods. During these times, due to the panel installation angle (southward and zero azimuth angle for the chosen site: Mansoura), sunlight strikes the rear side of the modules, resulting in zero energy generation^36^. Although shading losses from nearby and distant objects can range from 1% to 40%, they are not considered here as the system is installed on a building rooftop.

**Table 4:** The incident energy data for the chosen site.

	**Jan**	**Feb**	**Mar**	**Apr**	**May**	**Jun**	**Jul**	**Aug**	**Sep**	**Oct**	**Nov**	**Dec**	**Year**
**Horigental global (kWh**/$\hbox {m}^2$)	113.80	132.20	170.40	196.50	207.60	192.70	176.20	173.50	168.40	147.90	123	104.70	1906.90
**Horigental diffuse (kWh**/$\hbox {m}^2$)	39.30	42.70	68.40	76.10	98.10	102.90	103	97.90	71	63.70	39.10	39.40	841.60
**Extra-terrestrial (kWh**/$\hbox {m}^2$)	194.20	211.80	278.90	309.80	343.90	340.80	347.90	329.60	285.50	249.90	198.50	181.90	3272.60
**Clearness index (ratio)**	0.58	0.62	0.61	0.63	0.60	0.56	0.50	0.52	0.59	0.59	0.62	0.57	0.58
**Ambient temp. **($^o$**C)**	15.10	19.70	26.20	30.90	35.80	35.10	33.70	32.50	31.50	29.10	22.50	17.10	27.40
**Wind velocity (m/s)**	0.90	1.20	1.40	1.60	2.60	2.70	2.00	2.10	1.40	0.90	0.70	0.70	1.60

### Designing PV system

**Fig. 7:**
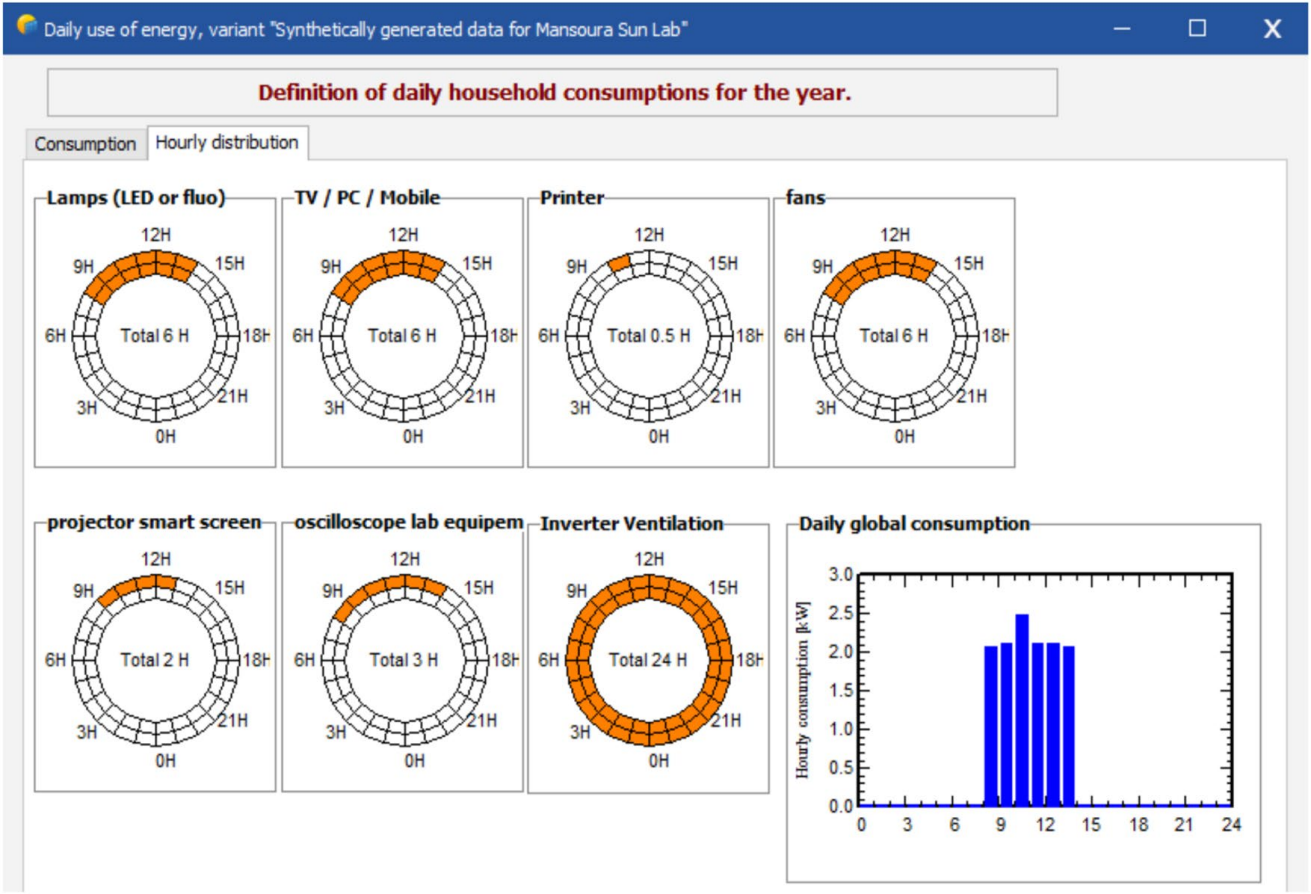
Load profile of Solar Energy Lab (total daily energy = 14.14 kWh, monthly energy = 424,4 kWh).

The total number of operating hours is assumed to be 5 hrs, where the peak power rating of the panel is considered to be 330 W. The operating factor is taken to be 0.75, and the peak equivalent is 0.88; i.e., sunlight available in a day is chosen to be only 8 hrs. Theoretical design of the solar system is done in the following few steps:

1. Load calculation: The average daily load consumption required for the Lab and its office and equipment operations is outlined in Table 5 and depicted in Fig. 7.
2. Battery specification: The specifications for the battery set used in the design of the system are detailed in Table 6.
3. Array (module): The specifications for the PV modules used in the system design are provided in Table 7.
4. Charge controllers: The universal controller 50 A MPPT Converter—built into the inverter-of 5 kW and 48 V—is used to design the stand-alone system having maximum charging and minimum discharging current, i.e., 50 A to 10 A.

**Table 5:** Load required in the Solar Energy Lab and office work.

**Sr. No.**	**Appliance**	**Power (W)**	**Number**	**Daily use** ** (hour/day)**	**Daily energy** ** required (Wh/day)**
1	Lamps (LED or fluo)	18	42	6	4536
2	Desktop/laptop	120	4	6	2880
3	Printers	250	3	0.50	375
4	Fan	40	8	6	1920
5	Projector-Smart screen	80	1	2	160
6	Oscilloscope-Lab equipement	80	12	3	2880
7	Inverter Ventilation	10	2	24	480
8	Stand by consumer	10		24	240
Total daily energy required					13471 Wh/day
Total monthly energy required					404.10 kWh/month

**Table 6:** Specification of battery set.

**Model**	**A512/200A**
Manufacture	Exide (Sonnenschein)
Battery in series	1
Battery in parallel	4
Total no of battery	12
Voltage	12 V
Battery pack voltage	48 V
Global capacity	600 Ah
Stored energy (80% DOD)	2.13 kWh
Total weight	804 kg
Number of cycles at 80% DOD	900
Total stored energy during the battery life	1,918 kWh

**Table 7:** Details of solar modules.

**PV module/Model**	**ND-AR330H**
Manufacturer/Year	Sharp/2020
Module power	330 Wp/37.10 V
Number of modules in series	2
Number of strings	4
Area	13 $\hbox {m}^2$
Sizing voltage	Vmpp (60 $^o$C) 32.50 V Voc(-10 $^o$C) 50.10 V
Max. operating power at 1000 W/$\hbox {m}^2$ and 50 $^o$C	2.41 kW

**Fig. 8:**
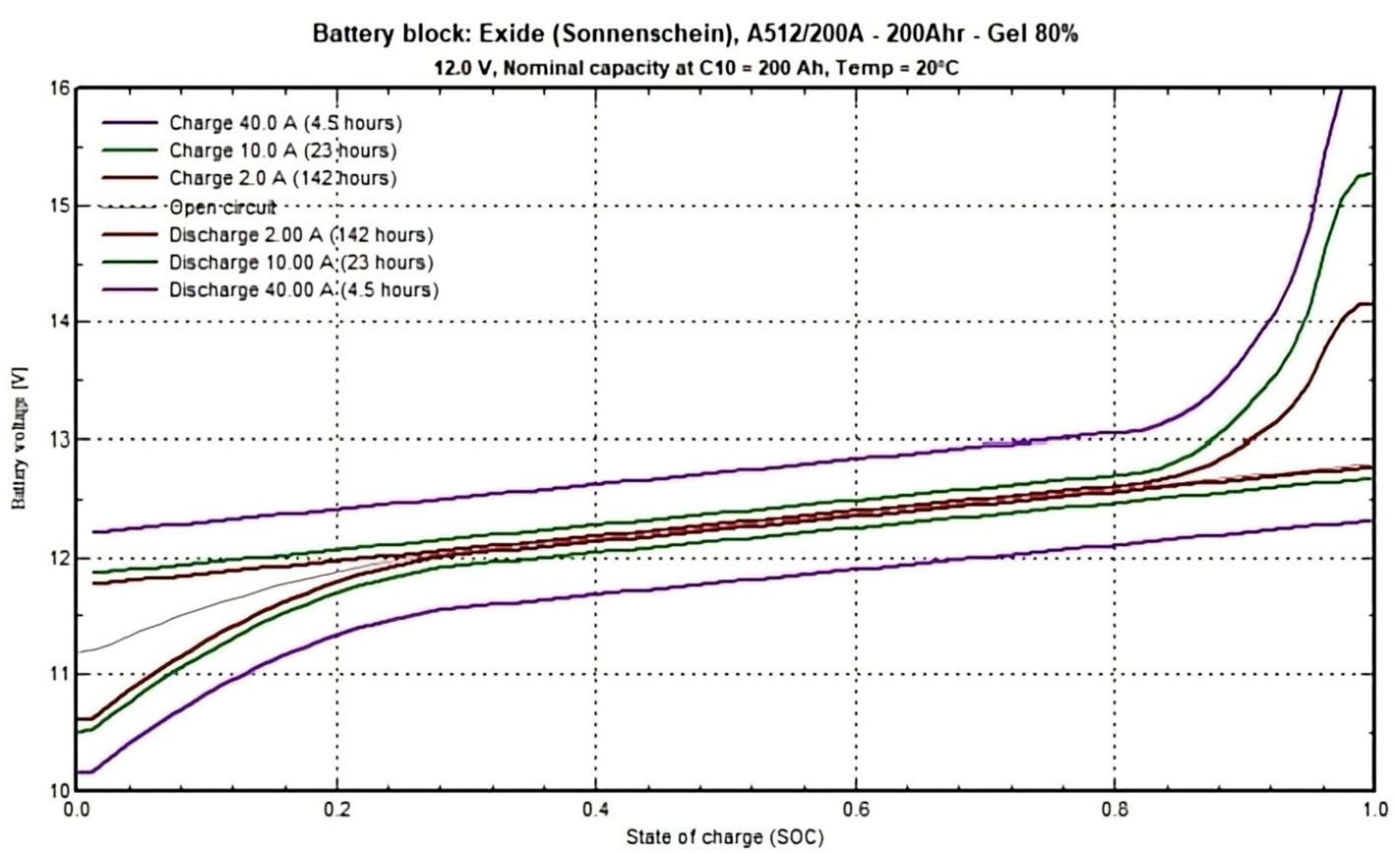
Battery block characteristics calculation by PVsyst.

Calculations for the entire load of the Lab are as follows:

1. Total PV power calculation: Since the modules are connected in series and parallel, the total output power of the array is calculated by using the following formula: Total power (W) = number of series modules $\times$ number of parallel strings $\times$ module power (W). Where the number of series modules = 2, the number of parallel strings (available at the Lab) = 4, and the module maximum power = 330 W, then the total power = 2 $\times$ 4 $\times$ 330 = 2640 W.
2. Daily energy production of the array: The energy produced by the array per day is calculated based on the total power of the array and the average daily solar irradiance hours at the selected location. where the daily energy production (Wh) = total power (W) $\times$ average daily solar irradiance hours. where total power = 2640 W and average daily solar irradiance = 5 hrs/day. Then, the daily energy production = 2640 $\times$ 5 = 13200 Wh/day = 13.20 kWh/day.
3. Battery capacity in watt-hours (Wh): The battery capacity, measured in Wh, is determined by converting ampere-hours (Ah) to watt-hours. This calculation serves to quantify the total energy storage capability of the battery as the following: Battery capacity (Wh) = battery capacity (Ah) $\times$ battery voltage (V), where battery capacity for one string (4 batteries in series) = 200 Ah. Battery capacity for three strings = 600 Ah, and battery voltage (4 batteries in series) = 48 V. Then, battery capacity = 600 $\times$ 48 = 28.80 kWh. The used battery characteristic is detailed in Fig. 8.
4. System Autonomy Calculation: Autonomy (days) = Battery capacity (Wh) / /average daily energy needs (Wh/day), where total battery capacity = 28.80 kWh and daily energy needs = 14.15 kWh. Then, autonomy =28.80/14.15 $\approx$ 2.04 days. This value determines how many days the system can operate using only the stored energy in the batteries, without any solar input. It’s a measure of how long the system can sustain the load during periods of no sunlight. Summary of the Results as following: Total power: 2640 W, daily energy production: 13200 Wh/day (or 13.20 kWh/day), battery capacity: 28800 Wh (or 28.80 kWh), and system autonomy: 2.04 days.

**Fig. 9:**
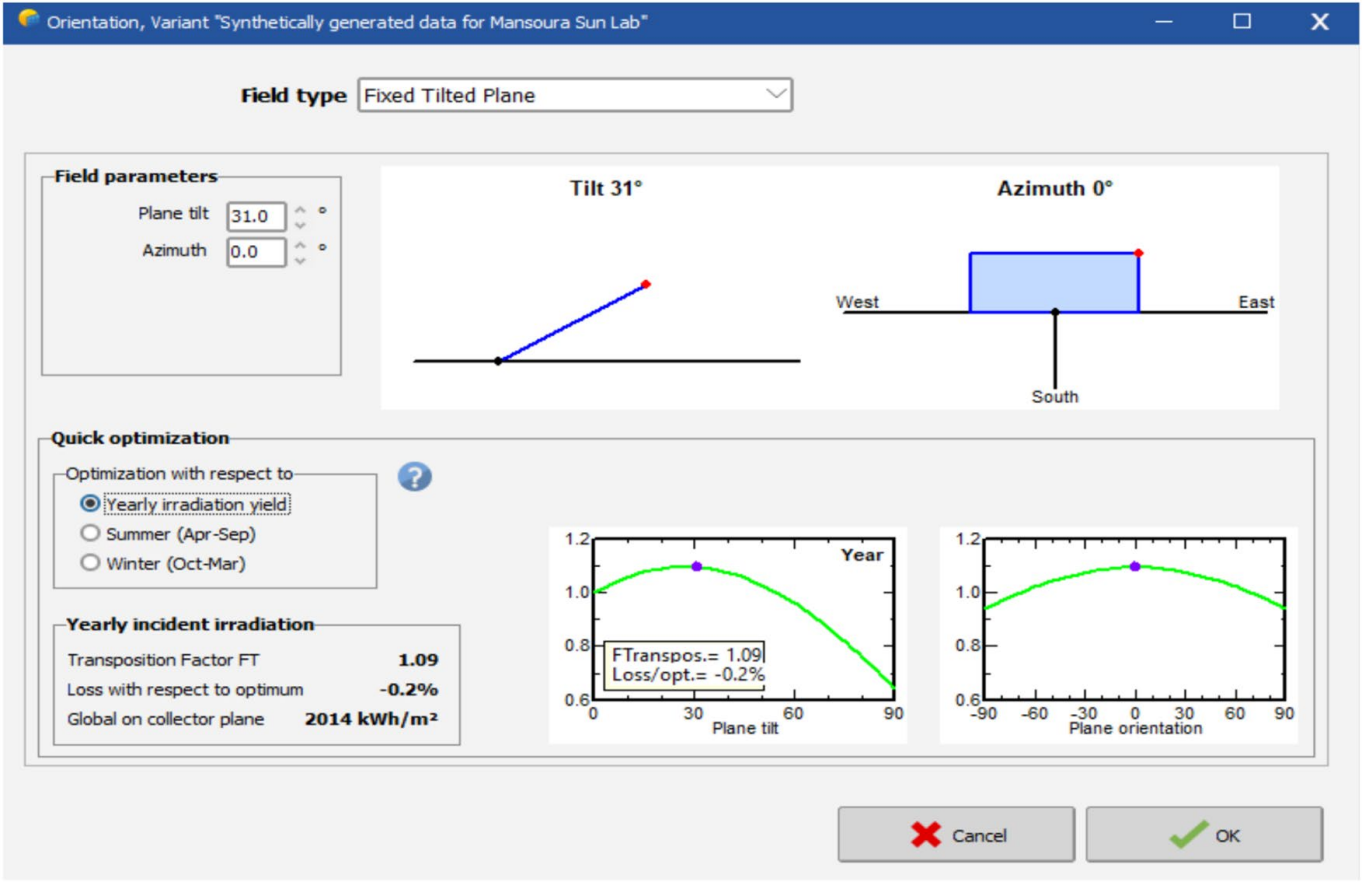
Solar irradiance calculation for the chosen site by using PVsyst.

Obiwulu et al.^37^ use simulations in identifying optimal tilt angle and radiation levels. Other theoretical models have been proposed to assess tilt angle performance across different latitudes north or south of the Equator. In this study, the PV panel structure is a fixed tilted plane of tilt $31^o$ and plane orientation azimuth (true south) $0^o$ as shown in Fig. 9. The optimization is done for the whole year with respect to optimum loss of zero percent, and the energy collector on the plane is 2014 kWh/$\hbox {m}^2$.

## Simulation

**Fig. 10:**
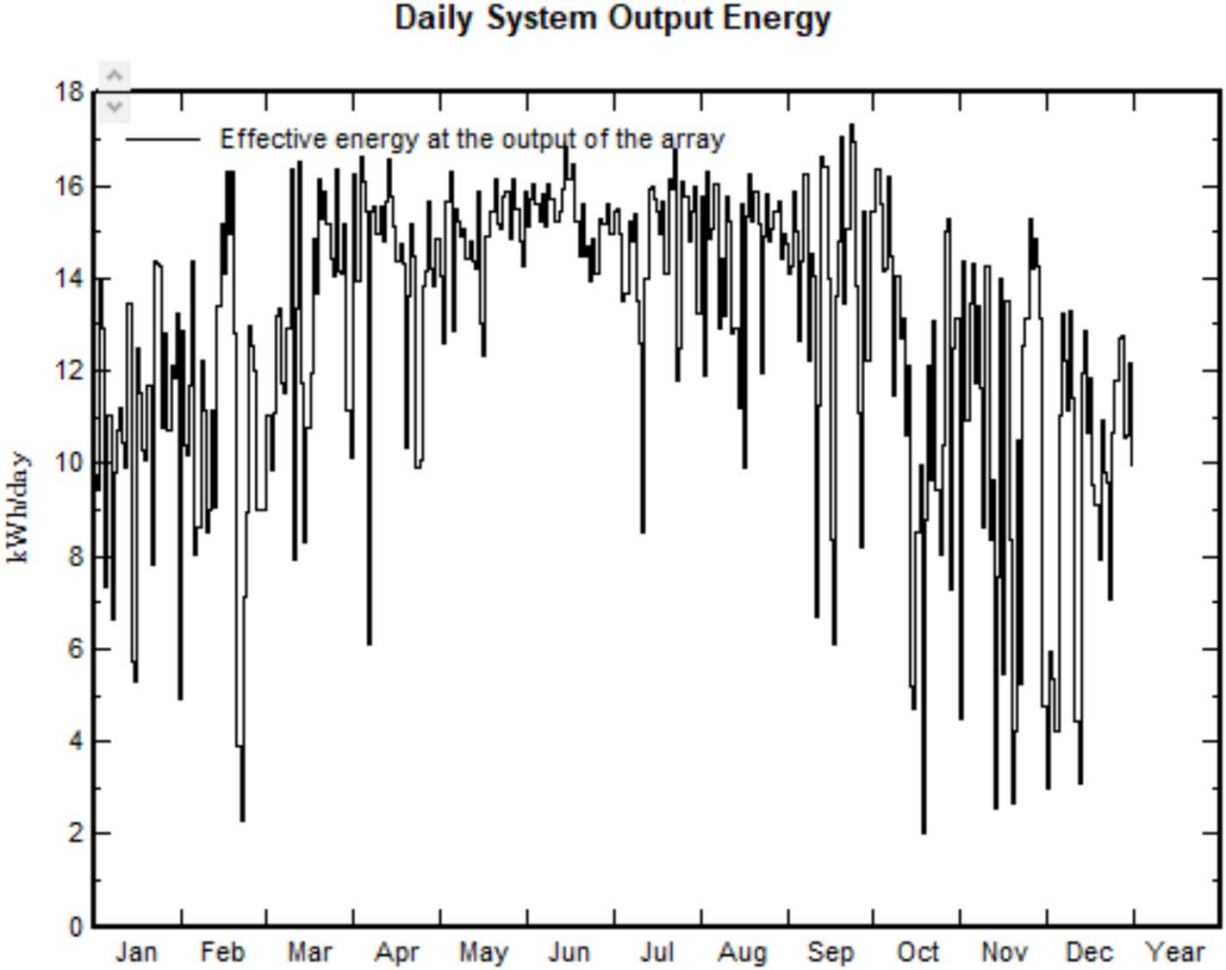
Daily system output energy.

The reliability of meteorological data, module models, and manufacturing specifications is uncertain in photovoltaic energy production. Properly installed rooftop solar panels, tailored to specific energy demands, offer a pathway to energy self-sufficiency for residential or small industrial use. This research provides valuable insights for future off-grid system design and operation, focusing on factors that influence system efficiency, such as material technology, energy generation methods, and manufacturing processes. Module behavior plays a pivotal role in defining system losses during simulation. The following results depict the distribution of yearly incident irradiation on a global collection plane and present data regarding monthly energy production relative to the system’s capacity. The analysis demonstrates the correlation between energy output and system losses, enabling a comprehensive understanding of the energy production dynamics throughout the year. In this study, PVsyst is employed to simulate the system’s performance, incorporating models for all components of the system to address various sources of losses. In this work, the performance ratio (PR), as an On-site production assessment, is a useful graphical tool for the system to indicate the yearly yield of energy production. The PR is seasonally dependent and must be found on precise irradiance data. To normalize short-term variability, the weather-corrected performance Ratio developed by NREL and implemented in the IEC 61724-1 standard compensates for seasonal temperature impacts, but not for other weather impacts such as irradiance level, wind, and soiling. All the presented results are based on the simulation results for the proposed site. Figure 10 illustrates the monthly energy production data from the solar power system, factoring in energy losses during production.

**Fig. 11:**
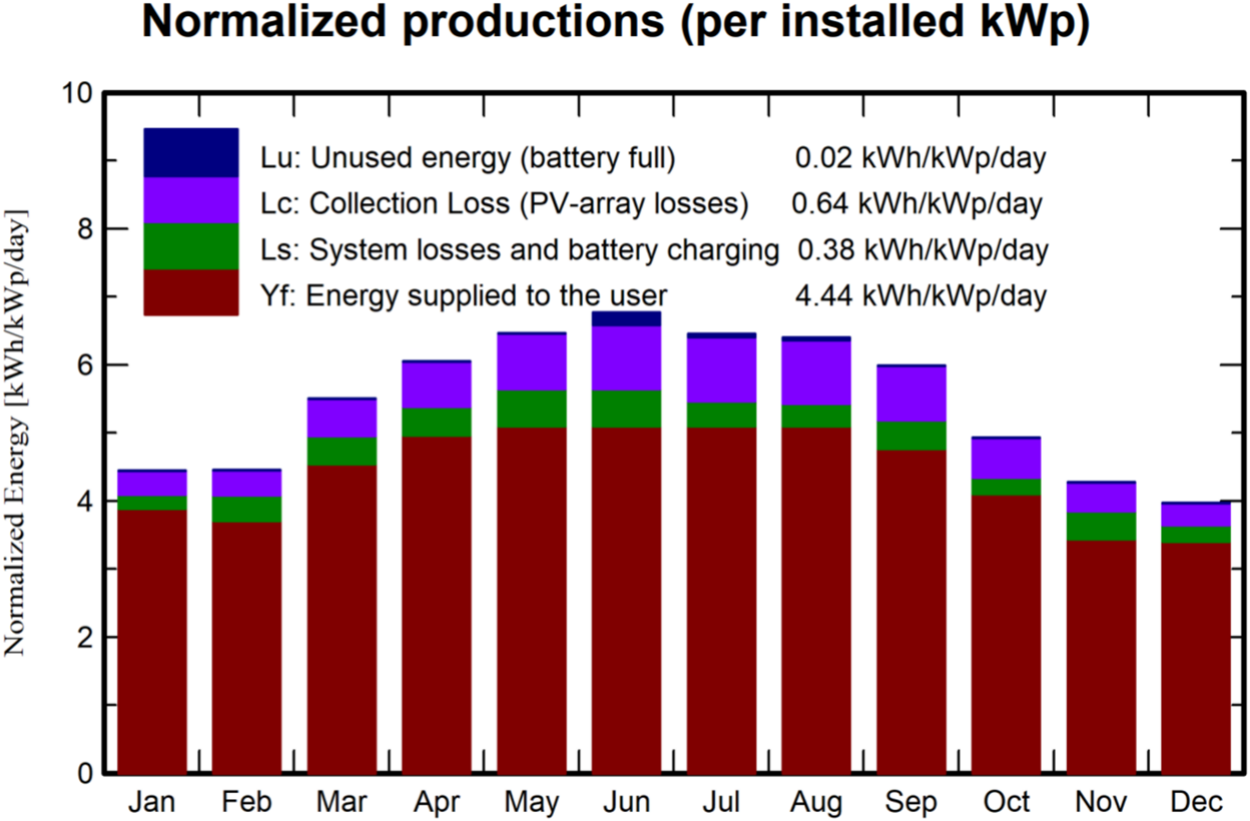
Graphical analysis of normalized energy distribution over the year.

The analysis, which incorporates weather records such as temperature and humidity at the selected locations, is depicted in Fig. 11. This figure also displays the monthly energy generation and the associated losses, revealing that the photovoltaic system’s average daily energy output over the year is 5.48 kWh/$\hbox {m}^2$. The highest energy generation occurs between May and August. On average, 4.44 kWh/kWp/day is supplied to the user, while array and battery charging losses account for 0.64 kWh/kWp/day and 0.38 kWh/kWp/day, respectively. Table 8 provides a summary of the annual energy balance for the off-grid system, indicating that 4279.80 kWh is delivered to the user annually. The simulation consistently demonstrates stable performance ratios throughout the year, as shown in Fig. 11.

**Fig. 12:**
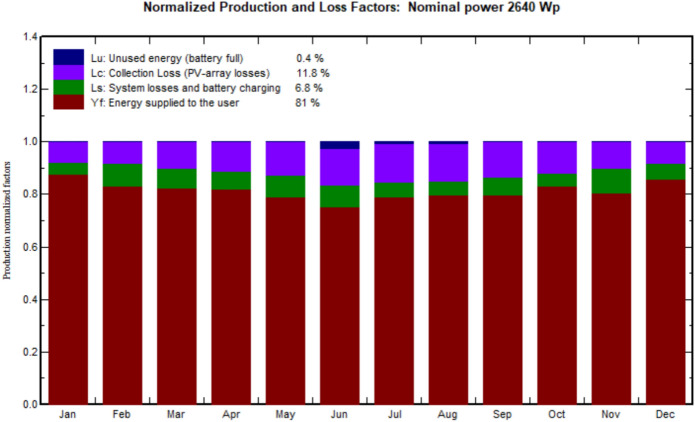
Monthly normalized productions with losses.

The normalized energy and performance ratio is shown in Fig. 12. The normalized production and performance ratio for every month has been depicted. Whereas the performance ratio is 81%, in which the system is working in good condition. The normalized production gives the three important parameters: 1- Collection loss (PV-array losses) is 0.67 kWh/kWp/day. 2- System loss (inverter, ...) is 0.38 kWh/kWp/day. 3- Produced useful energy (inverter output) is 4.44 KWh/KWp/day.

**Fig. 13:**
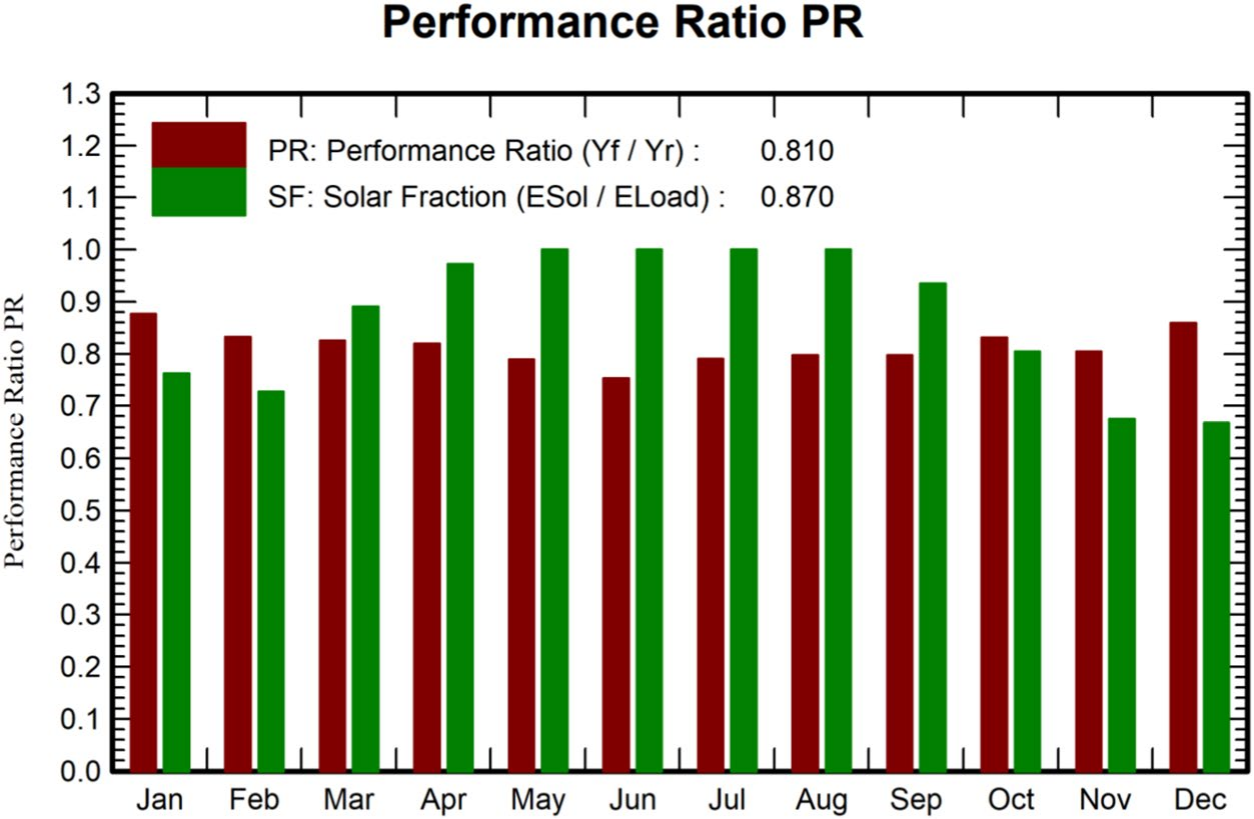
Performance ratio and solar fraction.

The analysis further reveals that the annual energy demand of the Solar Energy Lab is 4915.90 kWh, while the solar panels produce 4279.78 kWh, resulting in a power deficit due to various losses. The performance ratio (PR) and Solar Fraction (SF) of the system, as shown in Fig. 13, provide insights into system performance. The highest PR of 87.60% is recorded in January due to lower module temperatures, whereas the lowest PR of 75.34% occurs in June because of higher temperatures. The system achieves an annual average PR of 81%. Additionally, the solar fraction, which indicates the portion of energy needs met by solar energy, averages 87% annually, as detailed in Table 8.

**Table 8:** Yearly equalizations and fundamental results of off-grid frameworks.

	**GlobHor (kWh/**$\hbox {m}^2$)	**GlobEff (kWh/**$\hbox {m}^2$)	**E Avail (kWh)**	**E Unused (kWh)**	**E Miss (kWh)**	**E User (kWh)**	**E Load (kWh)**	**SolFrac (ratio)**
January	94.30	131.50	317.40	0	99.10	318.50	417.60	0.76
February	100.50	119	286.30	0.01	103	274.20	377.20	0.72
March	149.10	162.60	384.60	0	45.50	372.10	417.60	0.89
April	176.20	172.60	404.40	0	11.10	393	404.10	0.97
May	212.50	190	438.30	0.03	0	417.60	417.60	1
June	226.70	192	437.90	14.28	0	404.10	404.10	1
July	218.70	189.10	426.70	3.50	0	417.60	417.60	1
August	200.20	188	424.90	2.89	0	417.60	417.60	1
September	161.30	170.80	389.60	0	26.20	377.90	404.10	0.93
October	125.70	145.80	336.50	0	81.90	335.70	417.60	0.80
November	94.10	122.70	289.10	0.01	131.60	272.50	404.10	0.67
December	83.90	117.70	282.50	0	138.60	279	417.60	0.66
Year	1843.20	1901.70	4418	20.73	637.10	4279.80	4916.90	0.87

The PR, defined as the ratio of final system yield ($Y_{f}$) to reference yield ($Y_{r}$), is thoroughly explained in^38^. The software comprehensively analyzes all system loss factors during simulation, making it a critical tool for this study.

5 $$\begin{aligned} \begin{aligned}&PR = \dfrac{Y_{f}}{Y_{r}} \end{aligned} \end{aligned}$$

**Fig. 14:**
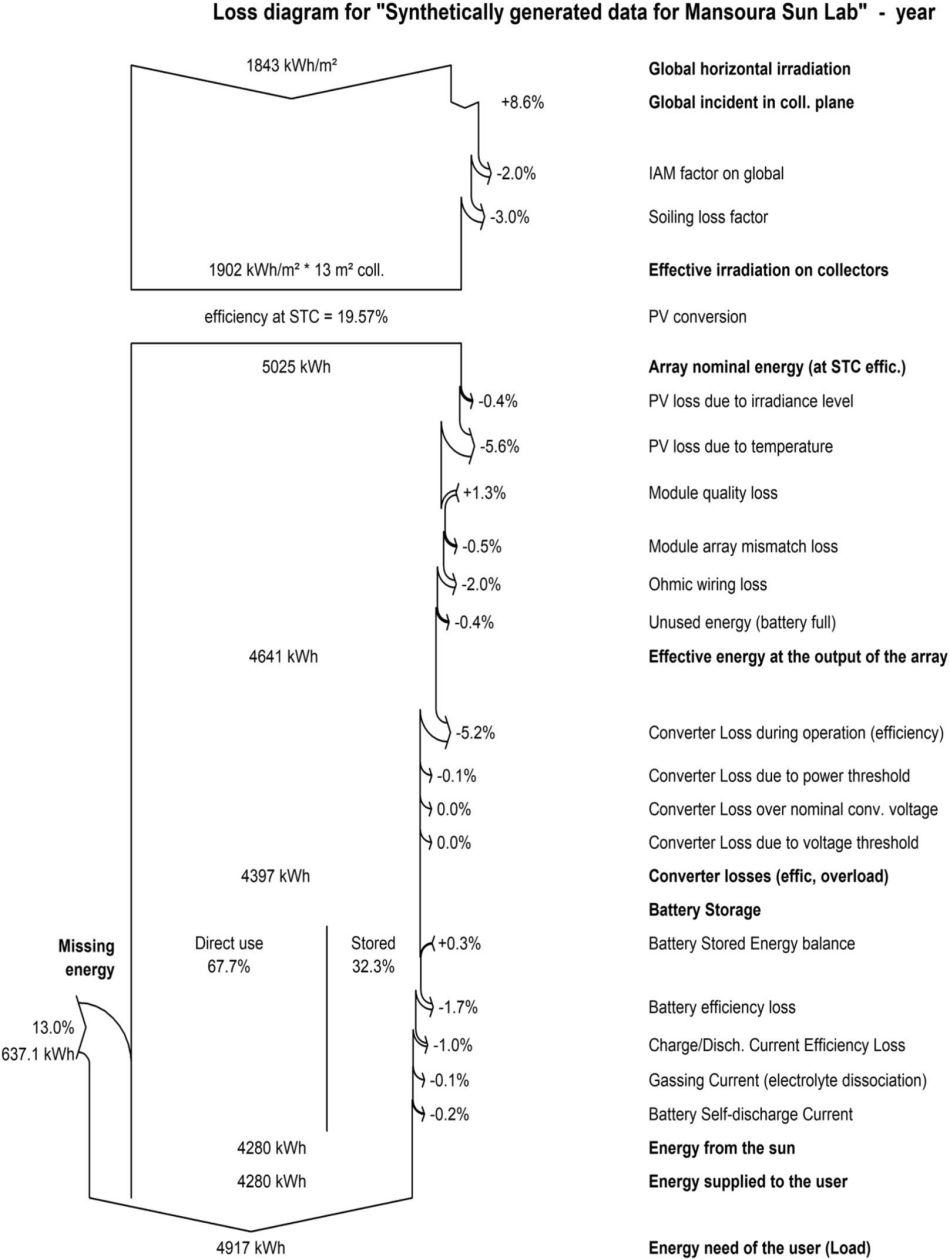
Arrow loss analysis of the proposed system.

Figure 14 with the distinct types of field losses within stand-alone photovoltaic systems step by step are illustrated in Fig. 14. The effective irradiation on collectors in this system is 1902 kWh/$\hbox {m}^2$ (with 8.60% global and incident irradiation in the collector plane) with the efficiency of 19.57%. In which the 1843 kWh/$\hbox {m}^2$ is falling in the chosen site to the system during one year. Due to array incidence loss and incidence angle modifier, incidence angle modifier (IAM) factor and soiling loss factor. Finally, 5025 kWh for array nominal energy at STC, and the rest of the energy is lost due to light-induced degradation (LID), mismatch loss, inverter loss during operation, and Ohmic loss. Yielding an energy need of the user (load) of approximately 4917 kWh.

Possible causes include undersized system or battery capacity, inaccurate and variable load profiles, high system losses due to the nature of the devices at the educational Lab, suboptimal issues, or unsuitable climate data, especially in this period. Further optimization will be addressed in future work.

**Fig. 15:**
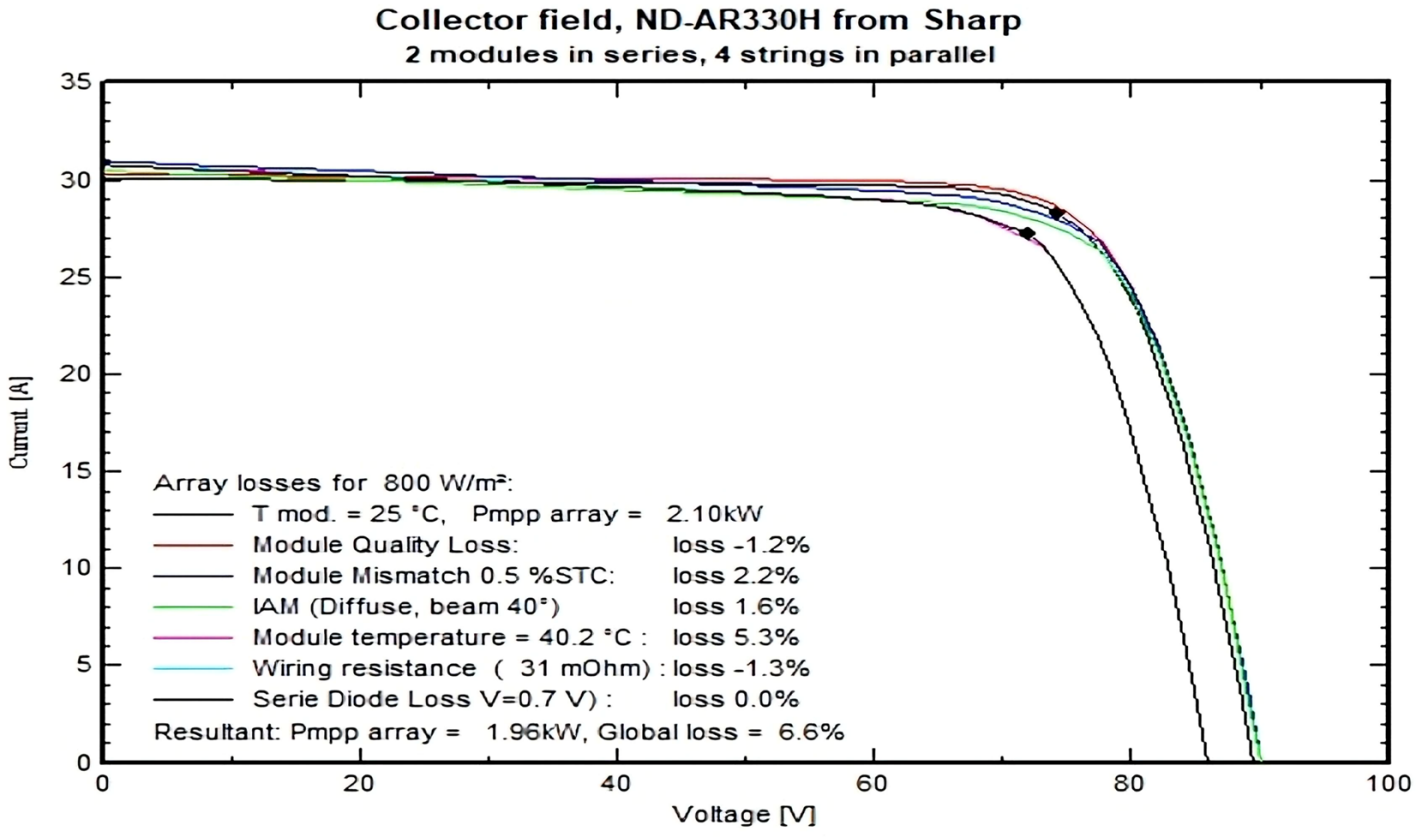
I–V curves with different losses for installed PV array.

Additional Insights: While the simulation results indicate promising energy outputs, there remains a shortfall in the energy available for user consumption due to inherent system losses. This highlights the importance of optimizing both array efficiency and energy storage solutions, especially in off-grid systems. In array voltage sizing, there are few conditions, such as the array maximum operating voltage must be below the maximum inverter operating voltage at the MPPT range. Also, the maximum array absolute voltage should not be more than the maximum system voltage. The V−I characteristics of PV systems with different losses is shown in Fig. 15.

**Fig. 16:**
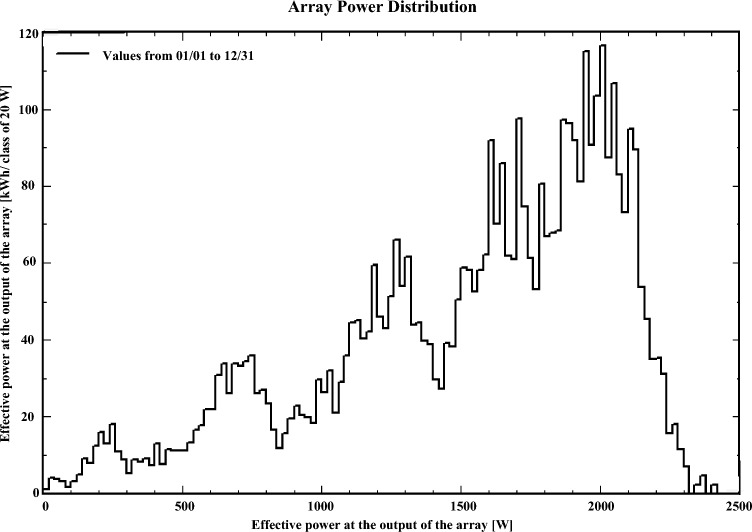
Daily energy and power available on inverter side of proposed stand-alone PV system.

**Fig. 17:**
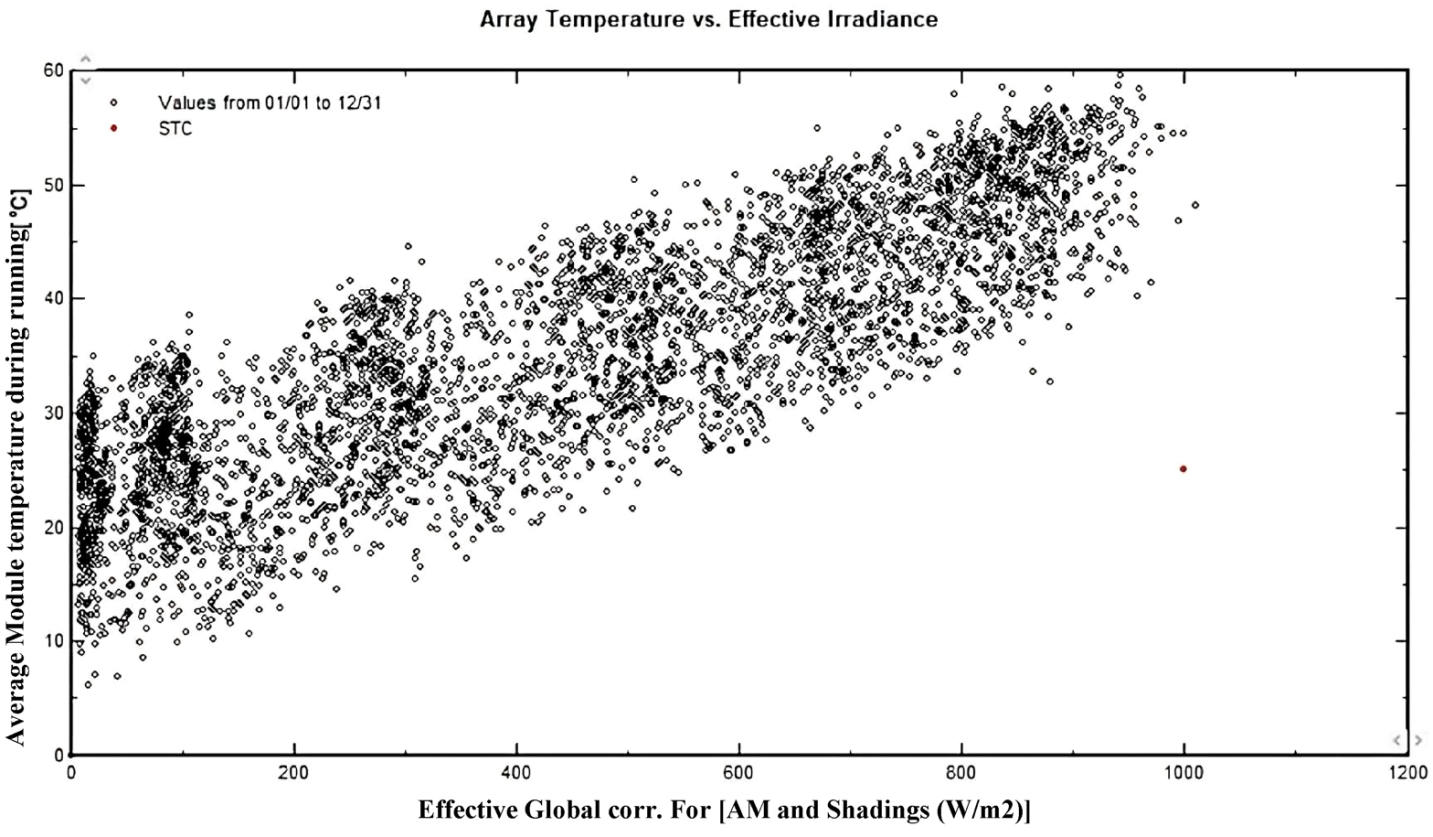
Array temperature vs. effective irradiance.

**Fig. 18:**
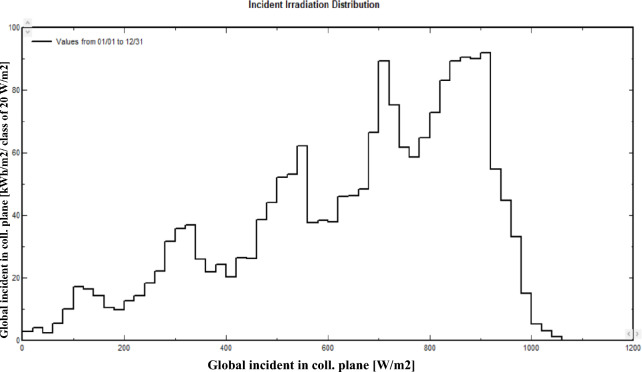
Incident irradiation distribution.

Variations in global irradiance significantly influence the performance of the solar system. As a result, the array power is shown in Fig. 16. Figure 18 illustrates the global irradiance distribution that resides on the collector plane of the 5 kW solar system, which is installed at Mansoura Solar Energy Lab. The hottest month in Mansoura is August, with an average temperature of the solar cells of approximately 65 $^o$C. The impact of the temperatures of the array throughout the year is shown in Fig. 17.

High temperatures are expected to significantly affect the open-circuit voltage, thereby reducing the output power of the array. In the PVsyst model, no losses occurred when horizontal irradiation was converted to global irradiation incidence. However, temperature derating, driven by hot and sunny conditions, resulted in a 5.60% loss. Additionally, the system experienced a 5.30% loss due to the converter, a 3% reduction from battery roundtrip inefficiency, and 13% of missing energy due to the probability of loss of load.

**Fig. 19:**
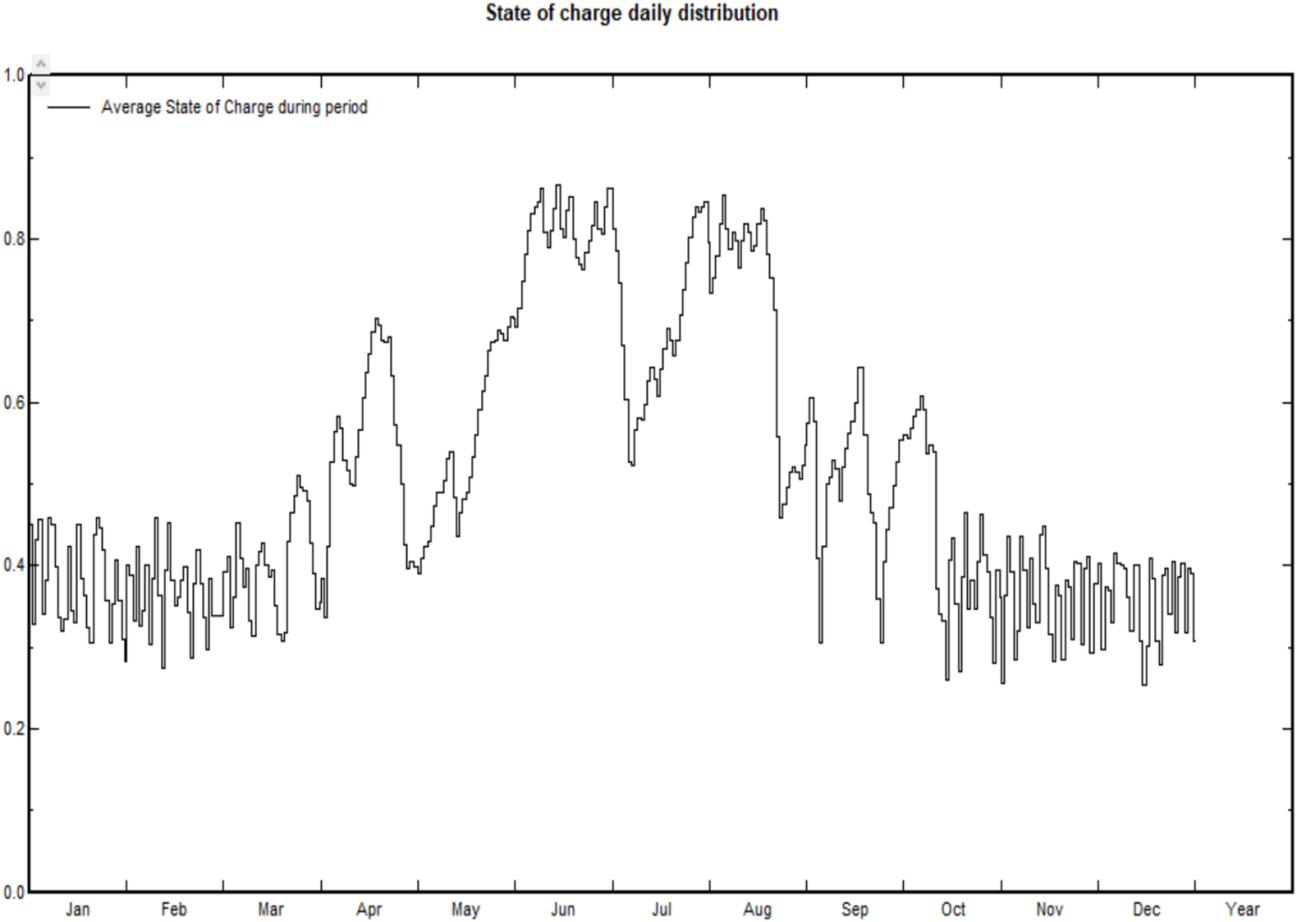
State of charge daily distribution.

The system is designed as an off-grid solution, allowing it to operate independently from the main power grid. The solar panels generate energy based on available sunlight, and while this energy is stored for use, the output can fluctuate depending on irradiance levels. Since solar generation is inherently variable and daily production cannot be precisely predicted, the system is equipped to manage these fluctuations. Figure 19 shows the average state of battery bank charging of 40%, and this is compatible with the above result for the design of the proposed system.

**Fig. 20:**
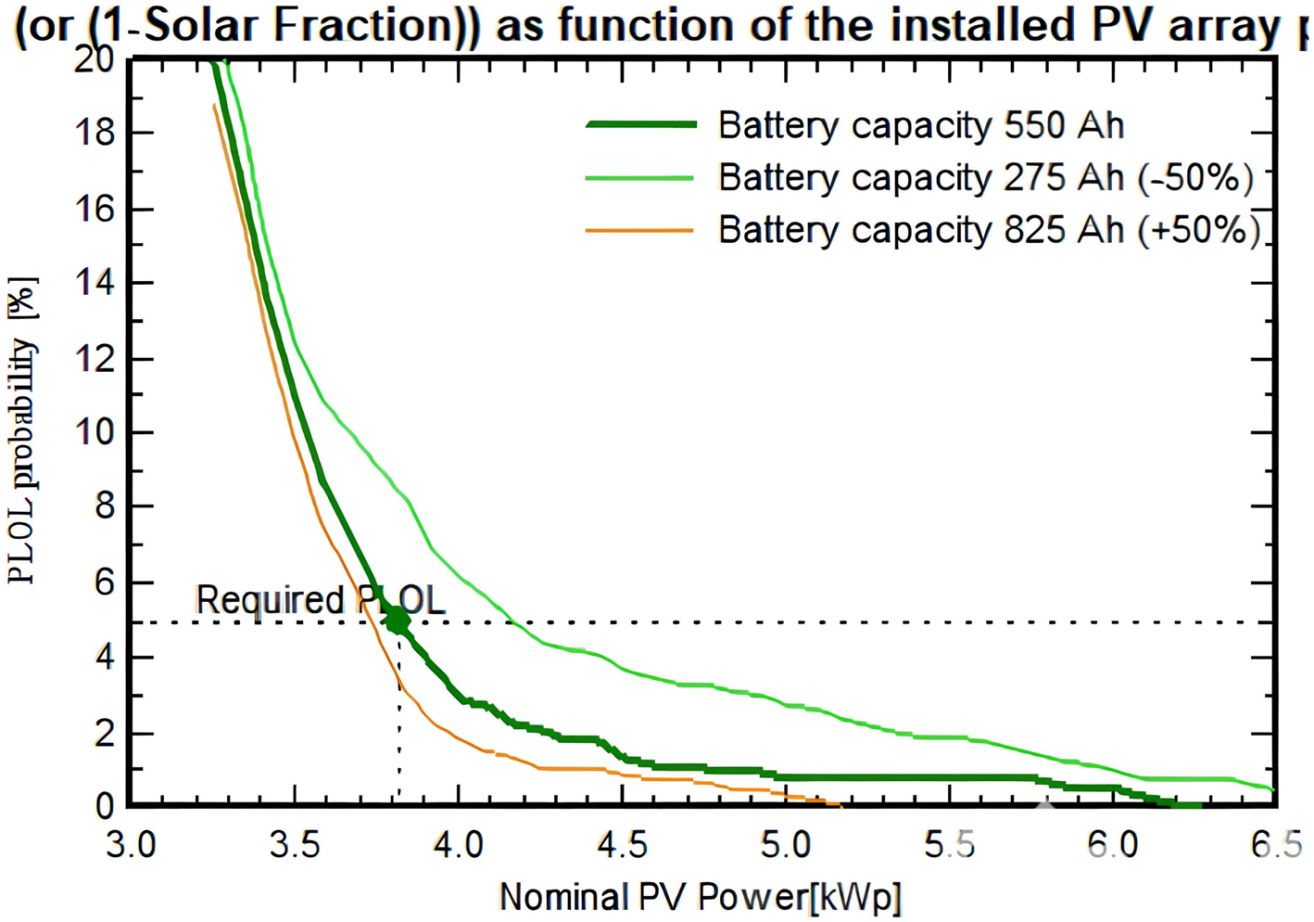
Solar fraction as function of output of installed PV array.

**Fig. 21:**
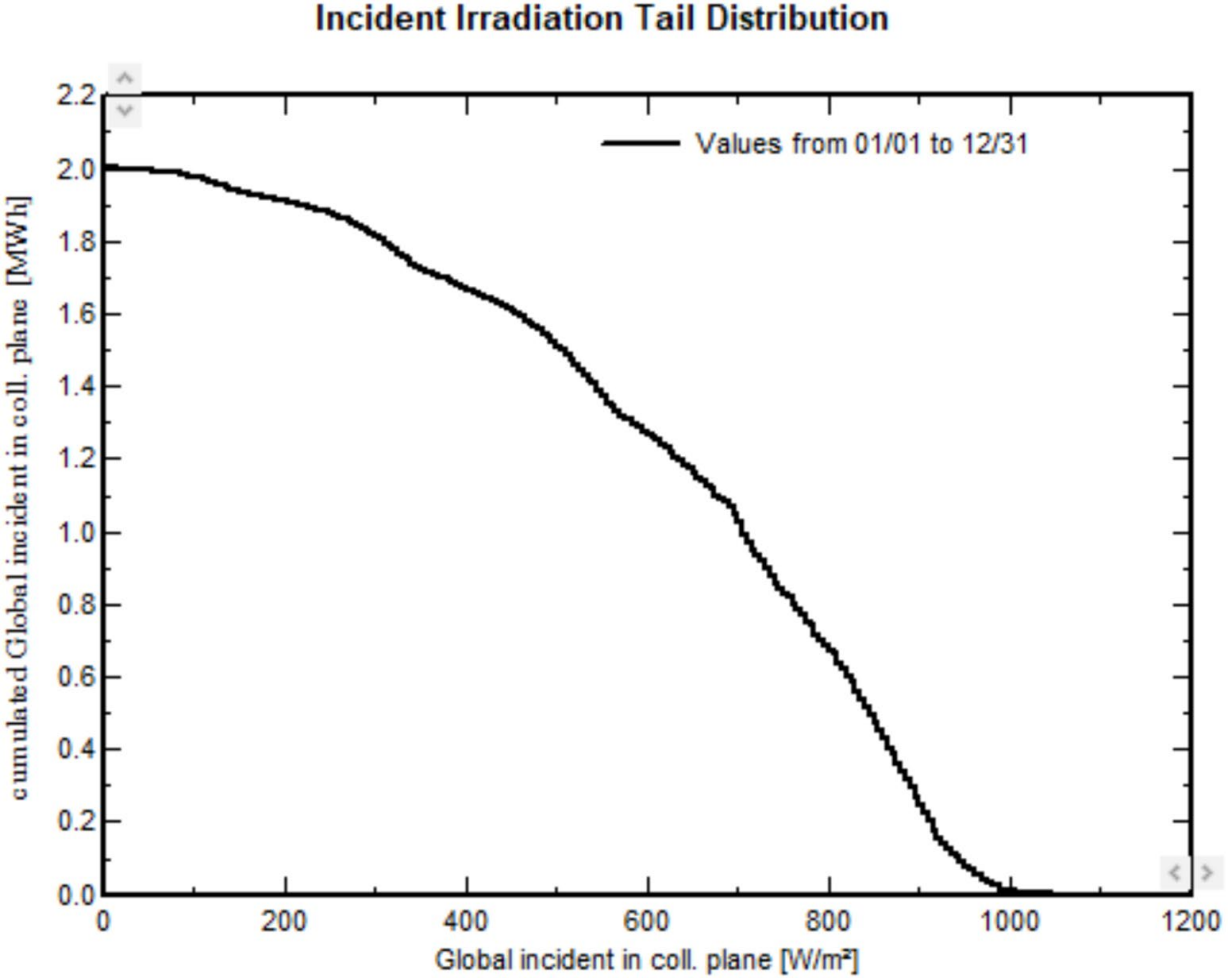
Incident irradiance tail distribution of proposed stand-alone PV system.

For 2.40 kWp, the required PLOL is found to be equal to 5%; the array detailed sizing tool shows the loss of load probability (PLOL) as a function of the installed array power as illustrated in Fig. 20. Figure 21 illustrates that the cumulative global incident irradiance on the collector plane decreases as the global incident irradiance increases. When the global incident irradiance reaches its peak value of 1000 W/$\hbox {m}^2$, the cumulative irradiance approaches nearly zero.

## Experimental validation

**Fig. 22:**
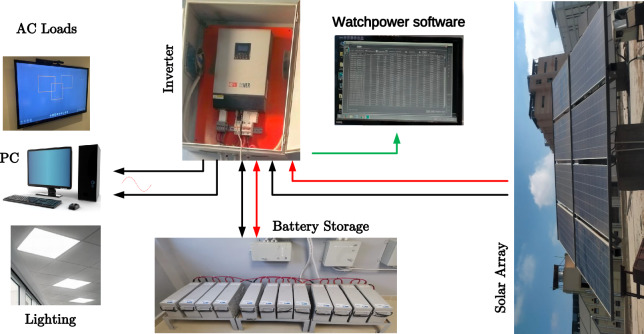
Block diagram of experimental setup of proposed stand-alone PV system.

The analyzed location shows high solar energy potential. It is ideal for implementing a photovoltaic system. PVsyst software can optimize the system design by adjusting layout, orientation, and capacity based on available solar resources. The solar irradiance data highlights stable energy conditions, enabling efficient energy production. PVsyst allows fine-tuning parameters like tilt angles and panel orientation to enhance output. This approach ensures high efficiency and promotes sustainable solar energy use. A prototype system is built and tested in real-time conditions, with power generation monitored using WatchPower software. The loads supplied by the stand-alone system at the Lab are the same as indicated by the Table 5. The experimental setup system at the Lab is shown in Fig. 22 which comprises the arrays, inverter, a battery package, connection boxes, PC-based Watchpower, and the Solar Energy Lab load. The inverter is supplied with its own built-in MPPT. The tilt angle of the photovoltaic arrays is set to align with the latitude (31^o^) to optimize solar energy capture for the chosen site: Mansoura.

**Fig. 23:**
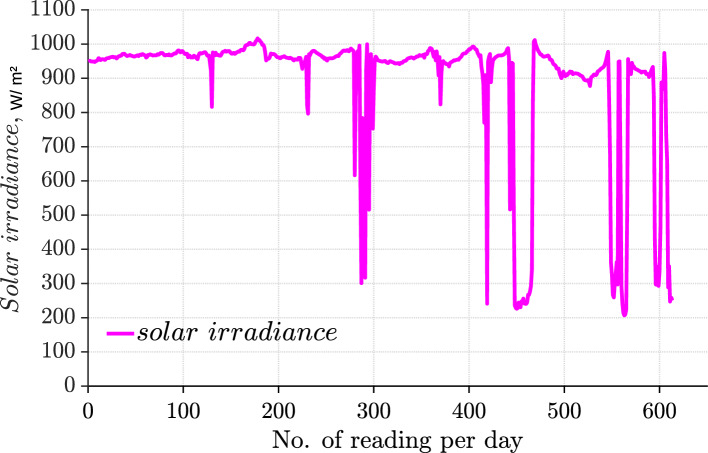
Experimental validation for solar validation (August measurement).

The received data from the inverter is analyzed by the WatchPower over the USB communication and employed for the research and the educational purposes. WatchPower aids in continuous data collection and optimization. The data confirms the consistent performance and peak load management. Combining the analysis performed with the PVsyst simulations supports the tailored design for local conditions. The pyranometer solar irradiance meter (SPM-1116SD) is employed to measure and record the solar irradiance intensity with an accuracy of (±10 W/m^2^). Figure 23 represents the natural stochastic solar irradiance during a moderately cloudy day in the summer: August. Besides, the measured solar power is more than 800 W under a partially cloudy day during midday in August on the inclined panel. The reading of power over three months is analyzed in the following section. The figures show only the daytime in which the system generates the power. The night intervals were deleted from all figures for simplification reasons.

**Fig. 24:**
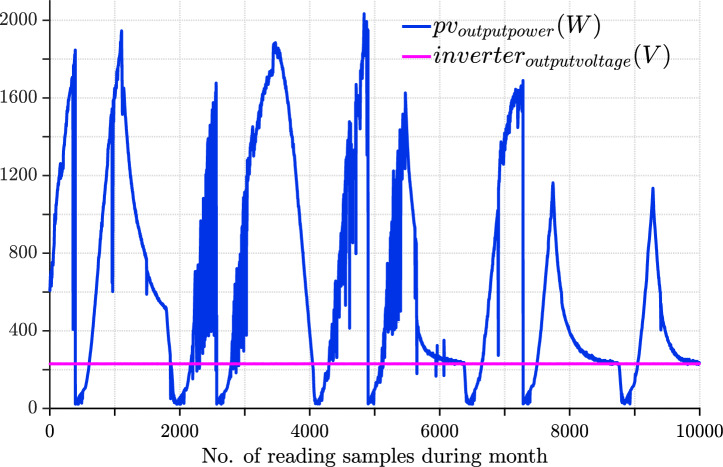
July readings showing daily PV output power (blue) and inverter output voltage (magenta). Average PV power for July was 675.47 W, with a maximum of 2113 W and total energy of 14.962 kWh.

**Fig. 25:**
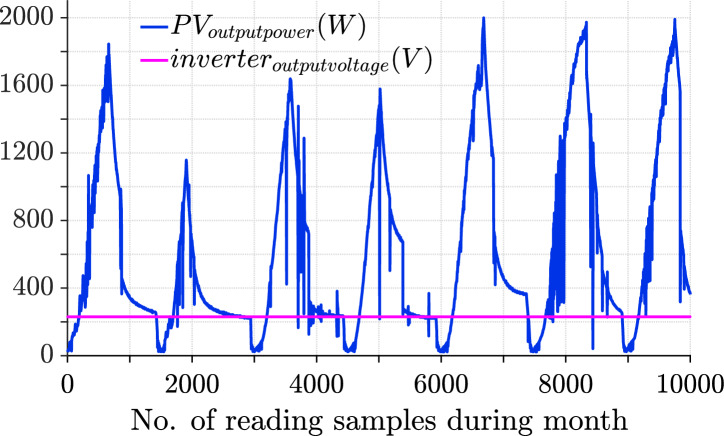
August readings showing daily PV output power (blue) and inverter output voltage (magenta). Average PV power for August was 727.63 W, with a maximum of 2317 W and total energy of 17.359 kWh.

The data presented in Fig. 24 indicates the energy output and performance of the photovoltaic system in July. The reading provides an overview of the photovoltaic system’s performance, highlighting key metrics such as average and maximum output power, as well as total energy generated. The average output power ($P_{pvavg}$) is approximately 675.47 W, indicating steady performance, while the system reached a maximum output ($P_{pvmax}$) of 2113 W during peak conditions. The total energy produced for the month of July ($P_{energy}$) is 1.4962 $\times$ $10^5$ Wh, demonstrating overall efficiency. These values suggest that the system performed reliably, with consistent energy generation and the capacity to handle peak energy demands effectively as indicated in Table 10. The August reading, which is presented in Fig. 25 represents key insights into the photovoltaic system’s performance, with slightly higher values compared to that of July. The average output power ($P_{pvavg}$) is approximately 727.63 W, reflecting an improvement in the system’s overall efficiency. The maximum output power ($P_{pvmax}$) reached 2317 W, indicating robust performance during peak conditions. Additionally, the total energy generated for the month ($P_{energy}$) amounted to 1.7359 $\times$ $10^5$ Wh, showcasing a higher cumulative output compared to the previous month. Overall, the system demonstrated enhanced energy production, maintaining consistent reliability while effectively managing peak power demands in August.

**Fig. 26:**
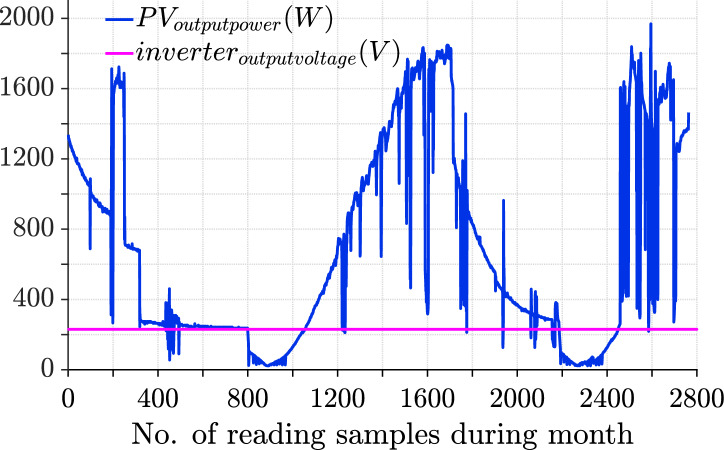
September readings showing daily PV output power (blue) and inverter output voltage (magenta). Average PV power for September was 678.28 W, with a maximum of 1969 W and total energy of 15.617 kWh.

Figure 26 represents September reading. The photovoltaic system’s performance slightly decreased compared to August. The average output power ($P_{pvavg}$) is 678.28 W, indicating a modest drop in efficiency. The maximum output power ($P_{pvmax}$) reached 1969 W, reflecting a reduction in peak performance compared to the previous month. The total energy generated for September ($P_{energy}$) is approximately 1.5617 $\times$ $10^4$ Wh, which is also lower than in August. Overall, the system maintained reliable energy generation but experienced a slight decline in both average and maximum output power during September. The values of power decreased due to the cloudy weather as expected before.

## Economic and environmental assessment

In modern engineering, economic and environmental evaluations are fundamental aspects of designing efficient and high-performance systems. Economic considerations have become a critical component of nearly every research effort.

### Economics perspective

There are many methods by which the cost of electricity generation can be calculated. One of the most commonly utilized methods is the so-called levelized cost of electricity (LCOE), average lifetime levelized generation cost (ALLGC), and levelized cost of generation (LCG)^5^. One of the most effective tools for evaluating the economic performance of various power generation systems is LCOE^11,39^. LCOE provides a comprehensive measure of the cost of electricity by dividing the total lifetime costs—including installation ($C_{\text {I}}$) and maintenance ($C_{\text {M}}$)—by the total energy produced over the system’s lifespan, while accounting for energy production degradation ($d$), as given by equation (6).

6 $$\begin{aligned} \begin{aligned}&LCOE = \frac{ \sum _{n=0}^{N} \frac{C_{\text {In}} + C_{\text {Mn}}}{(1 + i)^n} }{ \sum _{n=0}^{N} \frac{E_0 (1 - d)^n}{(1 + i)^n} } \end{aligned} \end{aligned}$$

where:

- $C_{\text {In}}$: Investment cost in year, $n$
- $C_{\text {Mn}}$: Maintenance cost in year, $n$
- $E_0$: energy produced in the first year (kWh),
- $d$: annual degradation rate (e.g., 0.005 for 0.5%),
- $$i$$: discount rate,
- $N$: number of years (project/system lifetime: 25 years).

To assess the economic feasibility of a 5 kW stand-alone system, the following procedures can be applied, assuming a lifetime of 25 years and an annual degradation rate of 0.5%. The financial cost parameters considered in the economic evaluation are presented in Table 9, including the installation and maintenance expenditure for the proposed system throughout its lifetime.

**Table 9:** Assumed costs of 5 kW stand-alone PV system: estimated in 2025.

**Component**	**Quantity**	**Unit Cost (USD)**	**Total Cost (USD)**
PV modules (330 W)	8	$150	$1,200
Batteries (200 Ah, 12 V)	12	$165	$1,980
Inverter (5 kW)	1	$1,000	$1,000
Installation & BOS	—	—	$500
**Total initial cost**			**$4,680**

By applying 0.5% annual degradation over 25 years, the total energy generated over the lifetime becomes:

7 $$\begin{aligned} E_{\text {total}} = \sum _{n=0}^{N} \frac{E_0 (1 - d)^n}{(1 + i)^n} \end{aligned}$$

where: $E_{\text {total}}$ is the total energy produced over the lifetime (kWh). The produced energy in the first year is about 4,418.01 kWh; then, the system is expected to generate 100,470 kWh over its lifetime, considering the degradation rate of 0.5% and neglecting the discount rate; *i*.

Since the system was installed at the Solar Energy Lab, the operation and maintenance costs are not counted and maintained by the Lab. The battery replacement will be every 7 years, so 3 times in 25 years (original batteries replaced twice). The battery’s future replacement costs are about $1,980 $$\times$$ 2 = $3,960. So, the total costs = initial ($4,680) + replacements ($3,960) = $8,640.

The levelized cost of energy (LCOE) is calculated as follows:

8 $$\begin{aligned} {LCOE}= & \frac{\text {total cost}}{\text {total energy}} \end{aligned}$$

9 $$\begin{aligned} {LCOE}= & \frac{8{,}640}{100{,}470} \approx \$0.086\text {/kWh} \end{aligned}$$

The calculations revealed that the system achieved an LCOE of 0.082 $/kWh, which can be decreased by optimizing the installed system and time schedule of operating hours at the Solar Energy Lab at Mansoura University.

### Assessment of $CO_{2}$ emission reduction for installed system

The transition to renewable energy sources (RESs) is essential to rescue the world from the negative effects of climate change, which is mainly due to $CO_{2}$ and particulate emissions. The photovoltaic systems offer a pollution-free source of power by reducing harmful emissions in electricity production. However, since they are quite inefficient, they require large surfaces in order to meet energy demands. While PV systems reduce emissions during operation, their manufacture and disposal currently emit greenhouse gases.

The carbon balance assessment for the photovoltaic system is computed based on Life Cycle Emissions (LCE), the quantity of $CO_{2}$ emissions by every component or energy output over its entire life cycle—from production, transportation, and installation to operation, maintenance, and disposal. The emission balance for an off-grid system installed at the Solar Energy Lab at Mansoura University, which is expected to produce approximately 4.42 MWh of electricity per year over a 25 years lifetime, accounting for a 0.5% annual degradation in performance. The system will replace an estimated 55.24 tons of $CO_{2}$ that would have otherwise been generated by the local grid, which emits 500 $gCO_{2}$ per kWh released to the atmosphere^40^.

**Table 10:** Key metrics, losses, and experimental validation summary for proposed system^11,27,30,40^.

**Aspect**	**Proposed method (PVsyst, experiment)**	**Range in literature**	**Remarks, Gaps**
Performance Ratio (PR)	0.81 avg (Sim), 0.78 (Exp June–Sept)	0.65–0.79	Above average due to optimal tilt angle and design; slightly lower in hot months (July).
Solar Fraction (SF)	0.87 avg (Sim)	0.70–0.95	Meets most of load demand; full SF (1.0) in May–August.
LCOE (USD/kWh)	0.082 (25 years, incl. battery)	0.10–0.15	Low due to local installation and minimal O&M costs.
System losses	$\bullet$ Temp loss: 5.6% $\bullet$ Inverter: 5.3% $\bullet$ Battery ineff.: 3% $\bullet$ PLOL: 13% (loss of load probability)	15–30% (total losses)	Losses align with typical ranges; deficit due to variable loads and limited storage.
Experimental accuracy	Avg. output: 727 W (Aug), Max: 2.32 kW	According to load demand	Exp. values confirm design; peak and average power stable across three months.
Deficit events / limitations	13% annual energy shortfall vs. load	5–15% in Labs without grid backup	Shows the need for further storage optimization or smart scheduling.

## Conclusion

This study presents a comprehensive design, simulation, and experimental validation of a stand-alone PV system for the Solar Energy Lab at Mansoura University. The proposed PV system, with a capacity of 2.64 kWp optimized using PVsyst software, demonstrates high efficiency in meeting an annual load demand of 4,279.78 kWh. Simulation results indicate an annual energy yield of 4,418.01 kWh, with a performance ratio (PR) of 81% and a solar fraction of 87%, confirming the system’s robust design. Economically, the system achieves a competitive levelized cost of energy (LCOE) of 0.082$ per kWh over its lifetime, while also contributing to environmental sustainability by offsetting an estimated 55.24 tons of $$CO_{2}$$ emissions compared to conventional grid supply. A critical contribution of this work is the experimental validation of the simulated performance. Over three months monitoring period, the system exhibited stable operation, generating between 149.6 and 173.6 kWh per month, with peak power outputs reaching 2.32 kW and an average daily power of 0.68 kW. To further enhance system efficiency and applicability, future research will focus on expanding PVsyst simulations with dynamic weather forecasting to improve energy yield predictions, conducting a detailed cost analysis of individual system components to refine economic feasibility assessments, and exploring hybrid configurations to enhance reliability for off-grid applications.

## Supplementary Information

Supplementary Information.

## Data Availability

The datasets used and/or analysed during the current study available from the corresponding author on reasonable request.
